# Insights from *Leishmania* (*Viannia*) *guyanensis *in vitro behavior and intercellular communication

**DOI:** 10.1186/s13071-021-05057-x

**Published:** 2021-10-28

**Authors:** Luiza O. R. Pereira, Cíntia S. Sousa, Hellen C. P. Ramos, Eduardo C. Torres-Santos, Liliane S. Pinheiro, Marcelo R. Alves, Patricia Cuervo, Gustavo A. Sierra Romero, Mariana C. Boité, Renato Porrozzi, Elisa Cupolillo

**Affiliations:** 1 Laboratório de Pesquisa em Leishmanioses, Instituto Oswaldo Cruz (IOC), Fundação Oswaldo Cruz (FIOCRUZ), Rio de Janeiro, Brazil; 2 Laboratório de Bioquímica de Tripanossomatídeos, IOC, FIOCRUZ, Rio de Janeiro, Brazil; 3 Present Address: Instituto de Saúde e Biotecnologia, Universidade Federal do Amazonas, Campus Coari, Amazonas, Brazil; 4 Laboratório de Pesquisa Clínica em DST-AIDS, Instituto Nacional de Infectologia Evandro Chagas, FIOCRUZ, Rio de Janeiro, Brazil; 5 Faculdade de Medicina, Universidade de Brasília, Distrito Federal, Brazil

**Keywords:** *Leishmania* (*Viannia*) *guyanensis*, Drug sensitivity, Treatment failure, Co-cultivation, Molecular mechanisms

## Abstract

**Background:**

Pentavalent antimonial-based chemotherapy is the first-line approach for leishmaniasis treatment and disease control. Nevertheless antimony-resistant parasites have been reported in some endemic regions. Treatment refractoriness is complex and is associated with patient- and parasite-related variables. Although amastigotes are the parasite stage in the vertebrate host and, thus, exposed to the drug, the stress caused by trivalent antimony in promastigotes has been shown to promote significant modification in expression of several genes involved in various biological processes, which will ultimately affect parasite behavior. *Leishmania* (*Viannia*) *guyanensis* is one of the main etiological agents in the Amazon Basin region, with a high relapse rate (approximately 25%).

**Methods:**

Herein, we conducted several in vitro analyses with *L.* (*V.*) *guyanensis* strains derived from cured and refractory patients after treatment with standardized antimonial therapeutic schemes, in addition to a drug-resistant in vitro-selected strain. Drug sensitivity assessed through Sb(III) half-maximal inhibitory concentration (IC_50_) assays, growth patterns (with and without drug pressure) and metacyclic-like percentages were determined for all strains and compared to treatment outcomes. Finally, co-cultivation without intercellular contact was followed by parasitic density and Sb(III) IC_50_ measurements.

**Results:**

Poor treatment response was correlated with increased Sb(III) IC_50_ values. The decrease in drug sensitivity was associated with a reduced cell replication rate, increased in vitro growth ability, and higher metacyclic-like proportion. Additionally, in vitro co-cultivation assays demonstrated that intercellular communication enabled lower drug sensitivity and enhanced in vitro growth ability, regardless of direct cell contact.

**Conclusions:**

Data concerning drug sensitivity in the *Viannia* subgenus are emerging, and *L.* (*V.*) *guyanensis* plays a pivotal epidemiological role in Latin America. Therefore, investigating the parasitic features potentially related to relapses is urgent. Altogether, the data presented here indicate that all tested strains of *L.* (*V.*) *guyanensis* displayed an association between treatment outcome and in vitro parameters, especially the drug sensitivity. Remarkably, sharing enhanced growth ability and decreased drug sensitivity, without intercellular communication, were demonstrated.

**Graphical Abstract:**

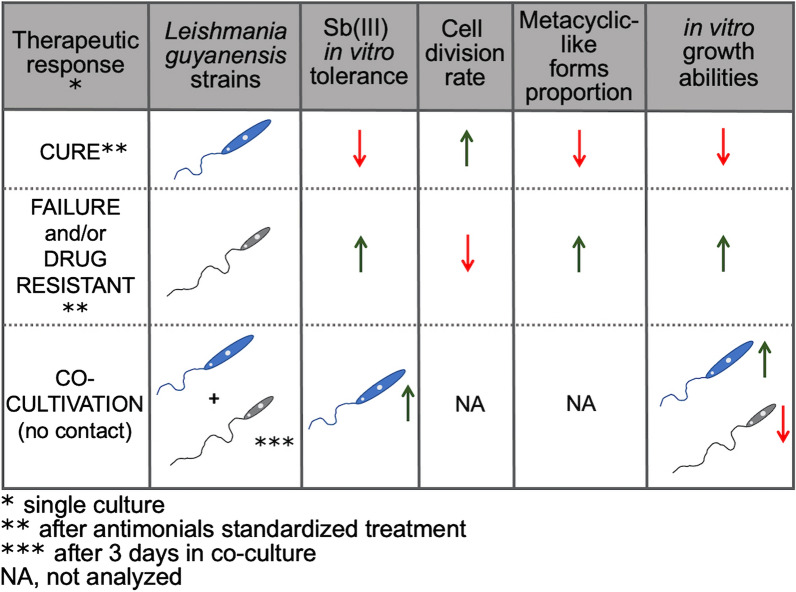

**Supplementary Information:**

The online version contains supplementary material available at 10.1186/s13071-021-05057-x.

## Background

Leishmaniasis is a complex of vector-borne diseases caused by parasites in the *Leishmania* genus and transmitted by phlebotomine sand flies. Almost 100 countries are affected by this disease worldwide [1]. A range of clinical manifestations can be observed, from a single ulcer, with spontaneous cure, to the visceral and potentially fatal form. Thus, American tegumentary leishmaniasis (ATL) comprises cutaneous, disseminated, mucous, and mucocutaneous presentations (when both lesions are present simultaneously). Mucous lesions usually develop several years after clinical cure of the primary ulcer. In Brazil, *Leishmania* (*Viannia*) *braziliensis* and *L.* (*V.*) *guyanensis* are the main etiological agents of ATL [2], and both species are already associated with the mucosal form of the disease [3]. Chemotherapy in leishmaniasis represents the first line of disease management. Amphotericin B, pentamidine, and miltefosine were recently incorporated as treatment agents for ATL, although antimonial-derived drugs have been the primary treatment alternatives for more than 70 years [4, 5]. However, antimonials display remarkable limitations, such as severe side effects and parasite resistance emergence, leading to treatment failure. Treatment failure has already been observed in many regions of the globe, such as Iran, India, and the Middle East [6–9].

Patient refractoriness is related to treatment failure that is multifactorial and depends on diverse drug, parasitic, and host features. Examples of host characteristics that may influence the outcome of leishmaniasis are mainly immunological, leading to stratified responses and differences between individuals [10]. Parasite features may also influence disease development, such as the species, differentially expressed parasitic virulence factors or molecular features, and the presence of endosymbionts [11]. Therefore, parasitic drug resistance represents one variable in the treatment failure equation. Thus, evaluating these components represents an enormous challenge and an interesting field for understanding parasite evolution and adaptation [12].

Pentavalent antimonial [Sb(V)] resistance (sodium stibogluconate, SSG-R), which has emerged in some *L.* (*Leishmania*) *donovani* subpopulations from the Indian subcontinent, has resulted in parasites with high infectivity, decreased in vitro Sb(III) sensitivity, and superior in vivo survival skills. These features may be related to the immunomodulatory capacity of SSG-R strains, leading to superior parasite loads and host multidrug resistance mutation 1 (MDR-1) manipulation reducing intracellular drug concentrations [13]. Therefore, host treatment could represent the driving force for the selection of the fittest *L.* (*L.*) *donovani* parasites [14]. Metacyclogenesis is a differentiation process that generates infectious forms that are transmitted by the vector bite [15]. Metacyclic forms are more abundant in late in vitro cultures and can be quantified by different methods. Their percentage was also higher in Sb(V)-resistant *L.* (*L.*) *donovani* clinical lines, suggesting more successful transmission, and thus greater fitness [16]. This evidence, along with increased proteophosphoglycan expression and superior in vivo virulence [17], indicated that *L.* (*L.*) *donovani* constitutes a particular example of increased fitness in a drug-resistant pathogen. Similar superior fitness in the less susceptible lineages was also observed in response to other anti-leishmanial drugs, including combinations of drugs [18]. On the other hand, for *L.* (*L.*) *major*, miltefosine-resistant strains have exhibited a distinct profile, with attenuated in vitro and in vivo virulence and typical survival indexes, regardless of the high proportion of metacyclic forms [19]. Similarly, there were no differences between a paromomycin-resistant *L.* (*L.*) *donovani* strain and its parent wild-type strain in terms of the growth rate, metacyclic percentages, and in vitro or in vivo infectivity [20].

Sb(V) is administered as a prodrug and is converted into the active and reduced form [Sb(III)] either in macrophages or amastigotes [21], by different activation mechanisms. A parasitic thiol-dependent reductase (TDR1) was identified and is able to reduce Sb(V) using glutathione [22]. Drug reduction may be also catalyzed by leishmanial antimonate reductase (ACR2) [23]. Finally, non-enzymatic reduction of Sb(V) is also mediated by both parasitic thiols, such as trypanothione, or macrophage-related molecules, such as glycyl-cysteine [24]. Once Sb(III) is inside the parasite, it reduces the adenosine triphosphate/adenosine diphosphate (ATP/ADP) ratio by reducing glycolysis and fatty acid β-oxidation beyond the consumption of intracellular thiol pools. Resistance mechanisms include decrease in drug activation capacity, variability in drug transport (influx/efflux), and trypanothione production and turnover (the key molecule for redox homeostasis in trypanosomatids) [4]. Recently, many of these features have been described in a laboratory-derived resistant *L.* (*V.*) *guyanensis* mutant, including reduced Sb(III) influx, increased energy-dependent Sb(III) efflux, and enhanced intracellular thiol levels [25].

The zoonotic context of ATL is very particular. Studies of different *Viannia* subgenus species have reported treatment failure [26–34] as well as antimony resistance emergence and related mechanisms [35–38]. However, the correlation between in vitro antimonial sensitivity and clinical outcome is conflicting; it is not observed in some cases [36, 39] but is documented for *L.* (*V.*) *braziliensis* strains [40], especially for isolates from atypical lesions [41, 42]. Additionally, many Sb(V)-resistant strains were isolated before treatment, mainly in *L.* (*V.*) *braziliensis* [36]. Together, these data indicate that more than drug resistance is behind treatment failure, supporting the hypothesis of epi-phenotype existence, originally proposed for *L.* (*L.*) *donovani*. This postulated that some adaptations not necessarily related to drug resistance might have been selected in the polyclonal population leading to drug decreased susceptibility and treatment failure [14]. One example relates to antimony-unresponsive strains and superior nitric oxide (NO) resistance associated to lower TNF-α levels [43]. Similarly, patients infected with NO-resistant strains displayed significantly larger lesions [44].

Nevertheless, the stress promoted by Sb(III) is an interesting approach for studying phenotypic modifications and its sharing through parasite–parasite communication. In this case, drug pressure represents a stressful environmental condition that might represent a selecting pressure source. Even though the amastigote is the developmental form exposed to the drug, it is very hard to study since it is intracellular. Thus, experiments from cultured Sb(III)-resistant promastigotes have been the main strategy to understand parasite drug resistance [37, 45]. Drug-related molecular mechanisms described for Old World species were also observed in antimony-resistant *L.* (*L.*) *amazonensis* [46]. Transcriptomic changes were observed in promastigotes of *L.* (*L.*) *amazonensis* strains adapted to in vitro Sb(III) exposure. Some genes were differentially expressed, either as a survival strategy or to induce cell death [47]. In *L.* (*V.*) *braziliensis*, *L.* (*V.*) *panamensis*, and *L.* (*V.*) *guyanensis*, Sb(III) pressure affected chromosomal somy, gene copy number, single-nucleotide polymorphisms (SNPs), and aneuploidy [48, 49].

Therefore, the present study was conceived with the clear notion that parasite drug-related mechanisms represent only part of the complexity of treatment failure. We hypothesized that the treatment outcome of patients infected with *L.* (*V.*) *guyanensis* and subjected to meglumine antimoniate (Glucantime^®^) therapy is correlated with the in vitro susceptibility of promastigotes to Sb(III). Additionally, we tested whether drug sensitivity affects metacyclogenesis and in vitro growth. Furthermore, we addressed the effect of inter-promastigote communication without cell contact on the sharing of these features.

## Methods

### Parasite cultures

Strains were acquired from the *Leishmania* Collection of the Oswaldo Cruz Institute (CLIOC, Brazil). Species identification and endosymbiont virus (LRV1) presence were determined during routine collection [50, 51]. Six *L.* (*V.*) *guyanensis* strains obtained from cutaneous leishmaniasis patients before Sb(V) treatment were selected according to treatment outcome: three from cured patients (IOC-L2335, IOC-L2370, and IOC-L2960) and three from patients with treatment failure (IOC-L2354, IOC-L2371, and IOC-L2372) (see Additional file 2: Table S1). The therapeutic scheme and the criteria for cure and failure determination are described in Additional file 2: Table S1, and were the same as those previously utilized by Torres et al. [52]. Promastigotes were grown at 25 °C in Schneider’s *Drosophila* medium (Vitrocell Embriolife, Campinas, SP, Brazil) containing 10% fetal bovine serum (FBS) (Cultilab, Campinas, S.P., Brazil), 100 UI/ml penicillin, and 50 μg/ml streptomycin (Merck, Darmstadt, Germany).

### Hamster (*Mesocricetus auratus*) infection and parasite isolation

All strains were reisolated from golden hamsters before assays. A total of 1 × 10^7^ parasites in 0.1 ml of sterile phosphate-buffered saline (PBS; Merck, Darmstadt, Germany) were inoculated in the posterior paw of golden hamsters. A swelling or lesion was observed in most animals. However, even if no signs were seen after 30 days, the animals were euthanized. Parasites were reisolated from subcutaneous biopsies in biphasic medium comprising Novy-MacNeal-Nicolle (NNN) medium with Schneider’s *Drosophila* medium, which was supplemented as described above, and were cryopreserved after a maximum of three passages.

### IC_50_ assays

A total of 2 × 10^6^ promastigotes were maintained in a flat-bottom 96-well plate (BD Biosciences, Franklin Lakes, NJ, USA) and were untreated or treated with trivalent antimony at concentrations from 2 to 60 µM for the cure group and 5 to 200 µM for the failure group. Negative controls and blanks without parasites were applied for each assay. Plates were incubated for 64 h at 25 °C, after which 20 μl of alamarBlue reagent (Thermo Fisher, Waltham, MA, USA) was added. The optical density was measured 8 h later in a SpectraMax spectrophotometer (Molecular Devices, San Jose, CA, USA) at wavelengths of 540 nm and 630 nm. The molar extinction coefficients of reduced and oxidized forms of the reagent and absorbance for both wavelengths were used to calculate the viability according to the manufacturer’s instructions and equations. The concentration effect curve was fitted for anti-promastigote testing via nonlinear regression, and the half-maximal inhibitory concentration (IC_50_) was determined using GraphPad Prism 7.0 software.

### Drug resistance selection and stability assessment along with culture and cryopreservation

The original strain IOC-L2335, which was derived from a cured patient, was maintained under drug pressure. The lineage from IOC-L2335 was maintained in regular biphasic medium with 10% FBS and supplemented with increasing concentrations of Sb(III) starting with 8 μM. The strain was transferred once each week to fresh media supplemented with an additional 4 μM of Sb(III) until the total concentration reached 104 μM. The whole process required 7 months and 24 passages. The resulting lineage, referred to as IOC-L2335R, was assayed for some of the analyzed parameters. The Sb(III) IC_50_ was assessed as described above at the end of the process and after cryopreservation.

### Growth curves

A total of 5 × 10^6^ parasites were added to 10 ml of Schneider's medium supplemented with 10% FBS. The number of parasites was evaluated by counting under an optical microscope with a Neubauer chamber at 8, 24, 48, and 72 h and as many 24 h-intervals as necessary until the declining phase was reached, corresponding to parasite death. Growth curves were also determined after cell synchronization for the selected strains. For this purpose, 3 × 10^7^ parasites/ml were inoculated in 25-cm^2^ flasks containing Schneider's medium supplemented with 10% FBS and 5 mM hydroxyurea [53]. Parasites were incubated for 8 h at 25 °C. After the treatment, aggregates (that corresponded to blocked cells) were removed by mild centrifugation. The resulting cultures were then centrifuged for 10 min at 3000×*g*. The supernatant was discarded, and the pellet was washed twice with 5 ml of Schneider’s medium to remove the hydroxyurea. The washed pellet was resuspended in 1 ml of Schneider's medium supplemented with 10% FBS, and the parasites were counted to determine the growth curve as explained above.

### Co-culture

In 24-well culture plates with Transwell^®^ inserts (BD Biosciences, Franklin Lakes, NJ, USA), a total of 1 × 10^6^ parasites with each profile (relapsed and cured) were added separately to the distinct compartments. A total volume of 1 ml of Schneider's medium with 10% FBS was used, with or without 8 µM Sb(III). Controls were prepared from cultures of the same strain in both compartments. Parasite numbers were determined by counting with a Neubauer chamber under light microscopy at 4, 8, 24, and 32 h until the declining phase of the growth curve was reached.

### Cell cycle analysis

After cell cycle synchronization, 1 × 10^6^ parasites were fixed with 2% paraformaldehyde (PFA) for 15 min at 4 °C. The resulting content was diluted in PBS containing 0.1% saponin, 5% fetal bovine serum, 1% bovine serum albumin, and 200 μg/ml RNAse and maintained at 25 °C for 30 min. Subsequently, 40 μg/ml propidium iodide was added, and the solution was incubated in the dark for 20 min at room temperature. A total of 10,000 events were acquired on a BD FACSCalibur™ cytometer (BD Biosciences, Franklin Lakes, NJ, USA). Data were analyzed in the Summit v4.3 program (Agilent, Santa Clara, CA, USA). All the above-cited reagents were purchased from Merck (Darmstadt, Germany).

### Promastigote morphological analysis

The analyses of procyclic (non-infective) and metacyclic-like forms were performed with stationary culture smears (from the fifth day of culture) that were fixed with methanol for 10 min, treated with 5 N HCl for 10 min, washed with water, and stained with Giemsa stain for optical microscopy visualization. The cell body length (L), width (W), and flagellum length (F) were measured in 100 promastigotes using the ImageJ program (Sun Microsystems, Santa Clara, CA, USA). Three independent biological replicates were assayed for each condition. The L × W and F/L values were calculated for each promastigote [54]. Each sample population was grouped into two subpopulations (including procyclic and metacyclic-like promastigotes). A *k*-mean cluster analysis (*k* = 2) was performed to determine the cutoff values for the two parameters (L × W and F/L). The percentage of metacyclic-like parasites was calculated for each sample using Excel (Microsoft Office 365 package). The average of the L × W was determined for each assay, and metacyclic-like forms were inferred if the L × W was lower than average and if F > L or F/L > 1.

### Statistical analysis

Prism 7.0 software (GraphPad Software, La Jolla, CA, USA) was used for statistical analysis. The Kolmogorov–Smirnov test was used to determine whether the values showed a Gaussian distribution. Appropriate analyses and post-tests were conducted and are described within the figure legends along with the *P*-values. Standard deviation is also shown in each figure or described in the tables.

## Results

### Therapeutic failure and in vitro-derived resistance phenotypes are associated with reduced sensitivity to antimonials

**Fig. 1:**
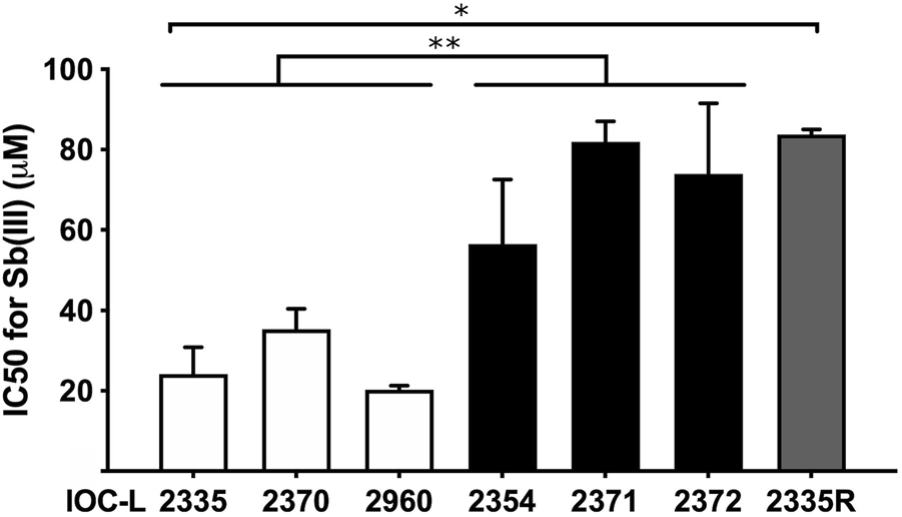
*Leishmania* (*Viannia*) *guyanensis* strains isolated from treatment failure patients exhibited higher IC_50_ values than strains from cured patients. The IOC-L numbers of each isolate are indicated in the figure; white bars represent cure-derived samples, black bars represent failure-derived strains, and the gray bar represents the resistant in vitro-selected isolate. Three biological replicates were assayed, and the error bars among them are represented. One-way analysis of variance (ANOVA) was used, followed by Tukey’s HSD (honestly significant difference) post hoc tests, to compare the IC_50_ means. *P < 0.01 and **P < 0.001

*Leishmania* (*V.*) *guyanensis* strains obtained from patients who were considered cured or who failed treatment will be referred to as C or F, respectively. In addition to the strains comprising the two treatment response groups—cure (IOC-L2335, IOC-L2370, and IOC-L2960) and failure (IOC-L2354, IOC-L2371, and IOC-L2372)—a lab-selected resistant strain (IOC-L2335R) with higher Sb(III) resistance than the parent strain was included in some of the assays. To obtain this lineage, the parent strain IOC-L2335C was maintained for consecutive passages under drug pressure until the new IC_50_ was comparable to the average of the failure-derived group Sb(III) IC_50_ (group mean = 70.78 and IOC-L2335R = 83.81). That increase corresponded to a resistance index of 3.5, relative to parent IOC-L2335C [37]. The achieved phenotype was stable in the absence of the drug, even after six passages and cryopreservation. The control without the drug was maintained throughout the whole resistance induction process, and a new IC_50_ assay was performed, which revealed no time effect. In conclusion, growth under Sb(III) pressure induced resistance, which was not affected by passaging or cryopreservation, even without the drug (see Additional file 4: Table S3). Strains from the failure group exhibited statistically higher IC_50_ values for Sb(III) than those from the cure group. Additionally, IOC-L2335R showed an increased IC_50_ compared to its parent sample (Fig. 1).

### *Leishmania* (*V.*) *guyanensis* strains derived from patients presenting different treatment responses exhibit diverse growth patterns

**Fig. 2:**
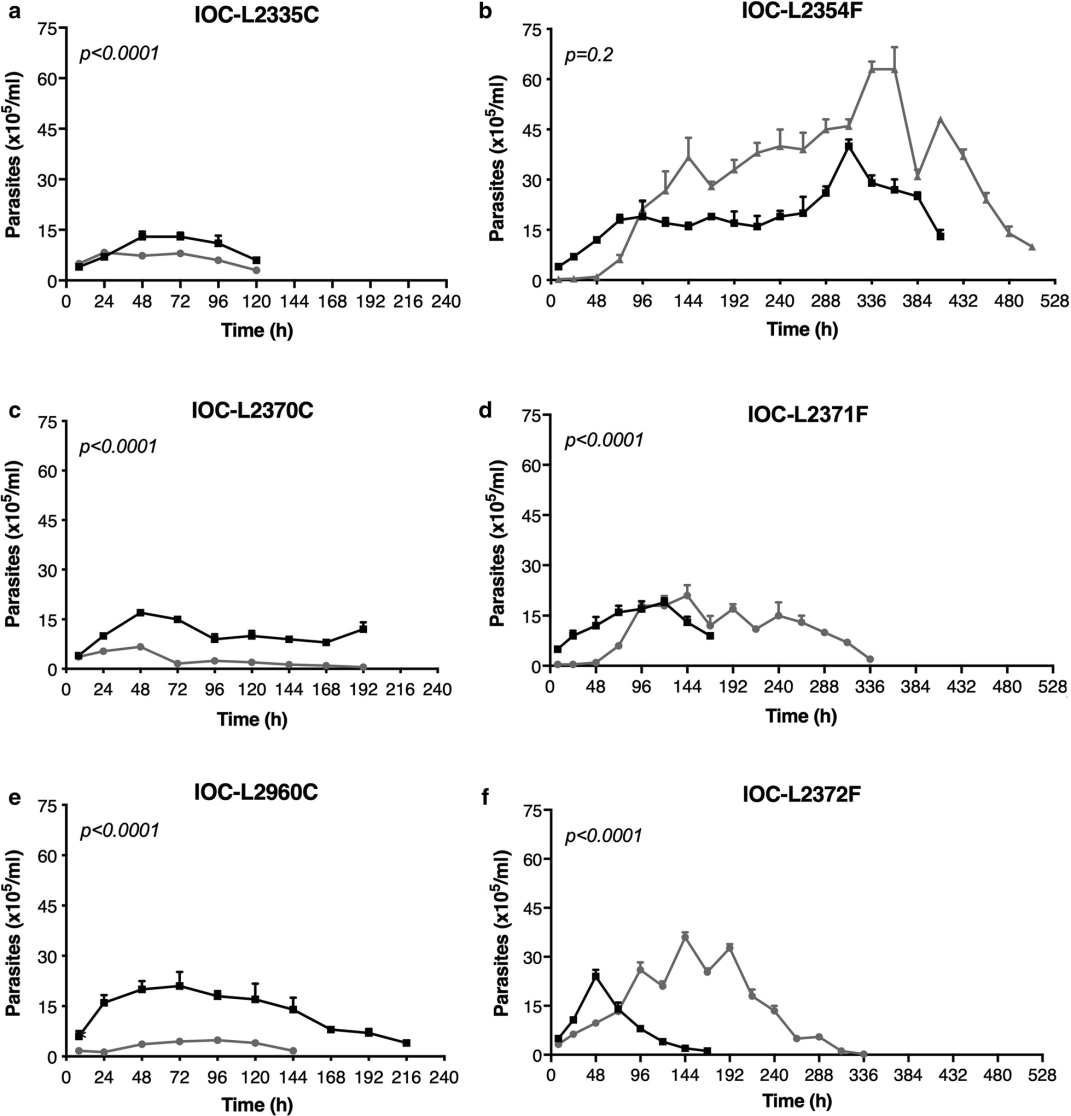
Antimonial exposure to isolates from patients with therapeutic failure boosted parasite growth. The growth curves of cure-derived (**a**, **c**, **e**) and failure-derived (**b**, **d**, **f**) isolates are shown. Black lines represent parasites maintained without Sb(III), and gray lines represent the number of parasite cells exposed to 8 µM Sb(III). Three biological replicates were assayed. Dots indicate means, and bars indicate the standard deviation. Pairwise *t*-tests with adjustments of the confidence level using Šidák's method were conducted for comparisons of the area under curve (AUC) of each isolate between treated and untreated samples. *P*-values for the comparisons are indicated in the figures. Table S2 with AUC comparisons can be found in supplementary material

**Fig. 3:**
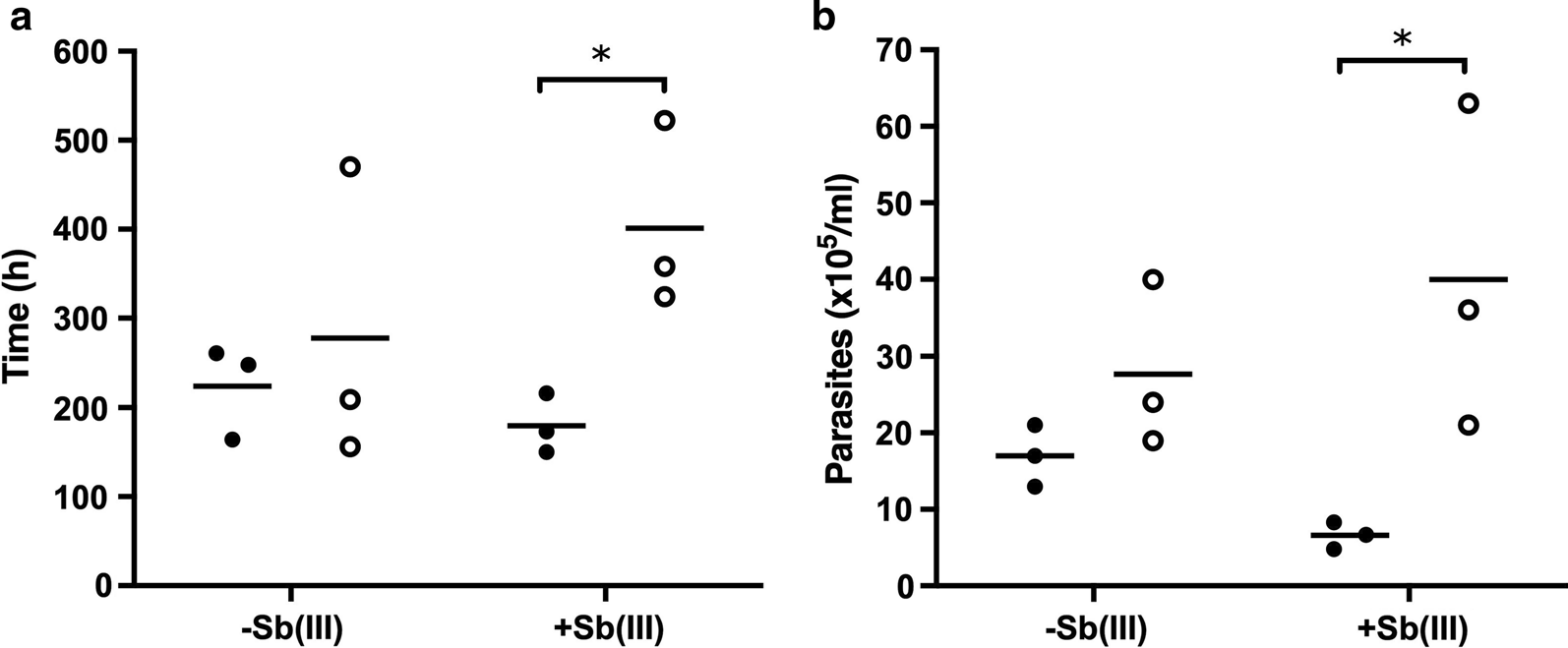
*Leishmania* (*Viannia*) *guyanensis* strains derived from patients with treatment failure exhibited higher growth than cure-derived strains, but only under drug pressure. According to the growth parameters, differences between cure and failure groups are shown as the maximum growth time in hours (**a**) and the maximum number of cells (**b**). Bars represent means, black circles represent the average of three biological replicates from isolates within the cure group, and white circles indicate the failure group. The values for the growth curves under 8 µM Sb(III) supplementation are indicated in the graph. Multiple *t*-tests were conducted for the cure and failure group comparisons [with or without Sb(III)]. *P < 0.05

To determine growth behavior, strains were maintained in culture with and without Sb(III) and were counted until low parasite densities were reached. Drug supplementation changed the growth curves significantly for all strains, except IOC-L2354 (failure-derived). The cure group presented similar growth profiles, exhibiting maximum cell amounts (without drug) and growth peaks at approximately 48–72 h. Conversely, the failure-derived strains showed distinct patterns. Under Sb(III) pressure, this group showed a notable lag phase during the first 48 h that was not observed for the cure-derived group; however, after this time point, this group exhibited high numbers of cells and an increased life span compared to cultures without Sb(III) (Fig. 2). To better illustrate these differences and between cure- and failure-derived groups, some additional data were extracted from curves and compared. The maximum number of cells and whole growth period, which was expressed in hours (Fig. 3), demonstrated that the group differences remained noteworthy only under drug pressure. The time when each curve reached zero, which corresponds to the culture death, was estimated. Since the exact time is hard to experimentally determine, these final time points were estimated using the linear part of the growth curve and Eq. (1), where *y* was zero, and *x* was the calculated time in hours when the parasites achieved the specific number:

1 $$y\, = \,ax\, + \,b$$

### The cure-derived strain exhibited the lowest growth under cell synchronization

**Fig. 4:**
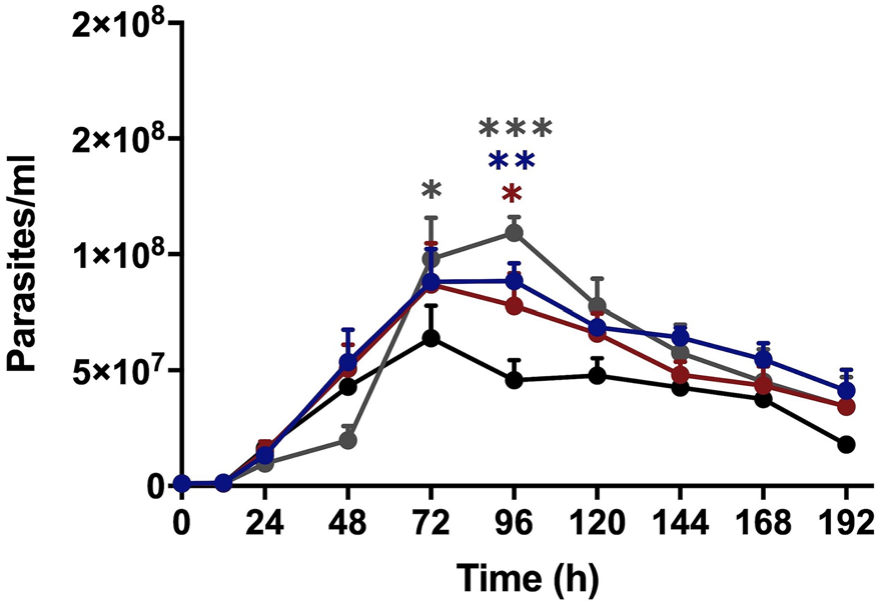
*Leishmania* (*Viannia*) *guyanensis* isolate derived from a patient cured after treatment (IOC-L2335C) exhibited the lowest growth compared to the treatment failure and in vitro-selected resistant isolates. Growth curves after cell synchronization for the cure strain IOC-L2335C are indicated in black. The curve of the failure-derived sample (IOC-L2371F) is displayed in gray. The resistant isolate selected in vitro (IOC-L2335R) is presented in dark blue in the absence of Sb(III) and in dark red when the media contained 8 µM Sb(III). Three biological replicates were assayed. Dots indicate means, and bars indicate the standard errors. Two-way ANOVA was used followed by Tukey’s HSD post hoc test for IOC-L2335C. *P < 0.05, **P < 0.01, ***P < 0.001

Growth curves were evaluated after cell synchronization with hydroxyurea [53]. This drug blocks the transition from G1 phase to S phase (Fig. 4). IOC-L2335C had the lowest number of parasites for most of the late time points. However, only the cell numbers at 96 h were significantly different compared with the control. Further undistinguishable growth patterns were observed.

### Cure-derived strain exhibited the lowest parasite counts and the lowest proportion of metacyclic-like forms

**Fig. 5:**
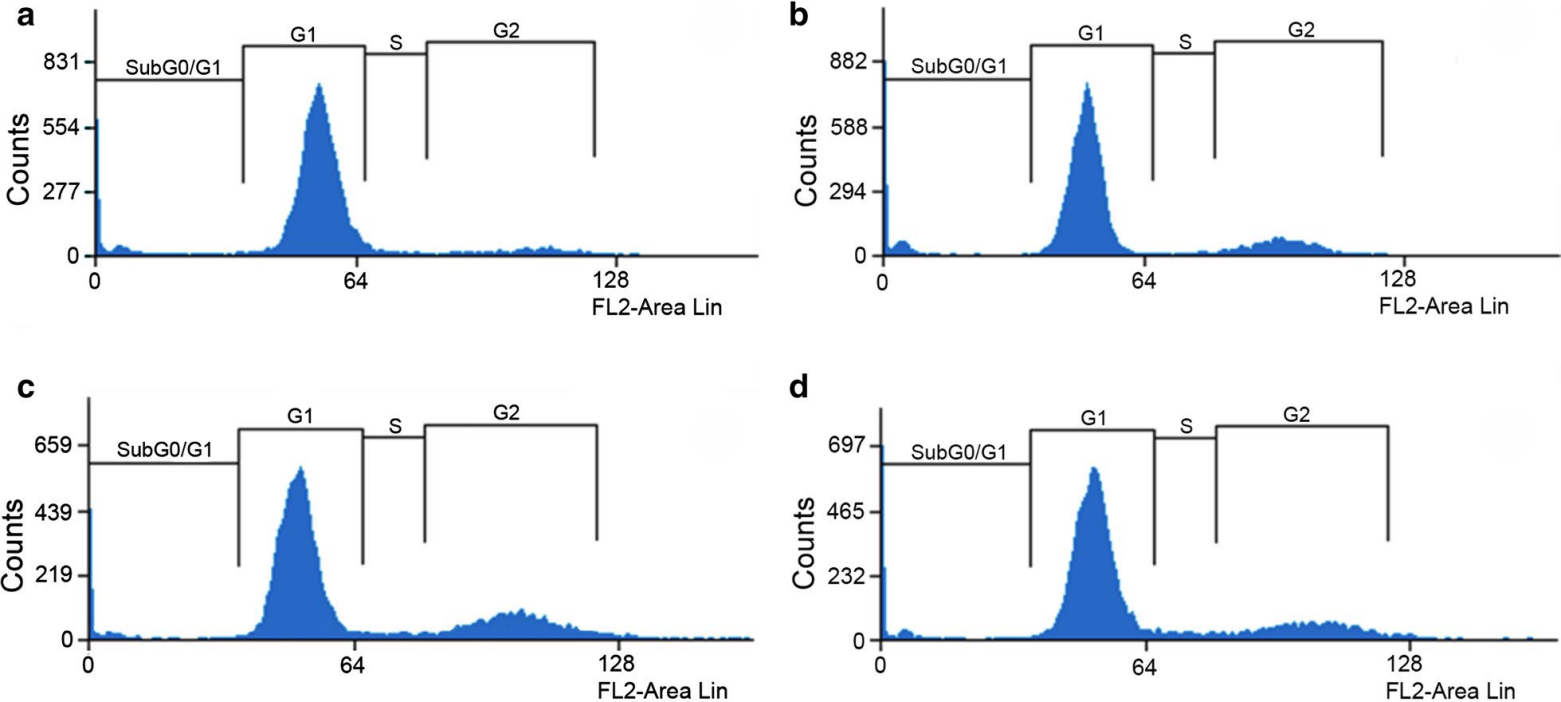
Propidium iodide staining revealed the cell cycle phases of cultures at the stationary phase (fifth day of culture) for all isolates. The graphs correspond to the IOC-L2335C (**a**), IOC-L2371F (**b**), IOC-L2335R + Sb(III) (**c**), and IOC-L2335R-Sb(III) (**d**) strains. A representative figure of three different biological analyses is shown

**Fig. 6:**
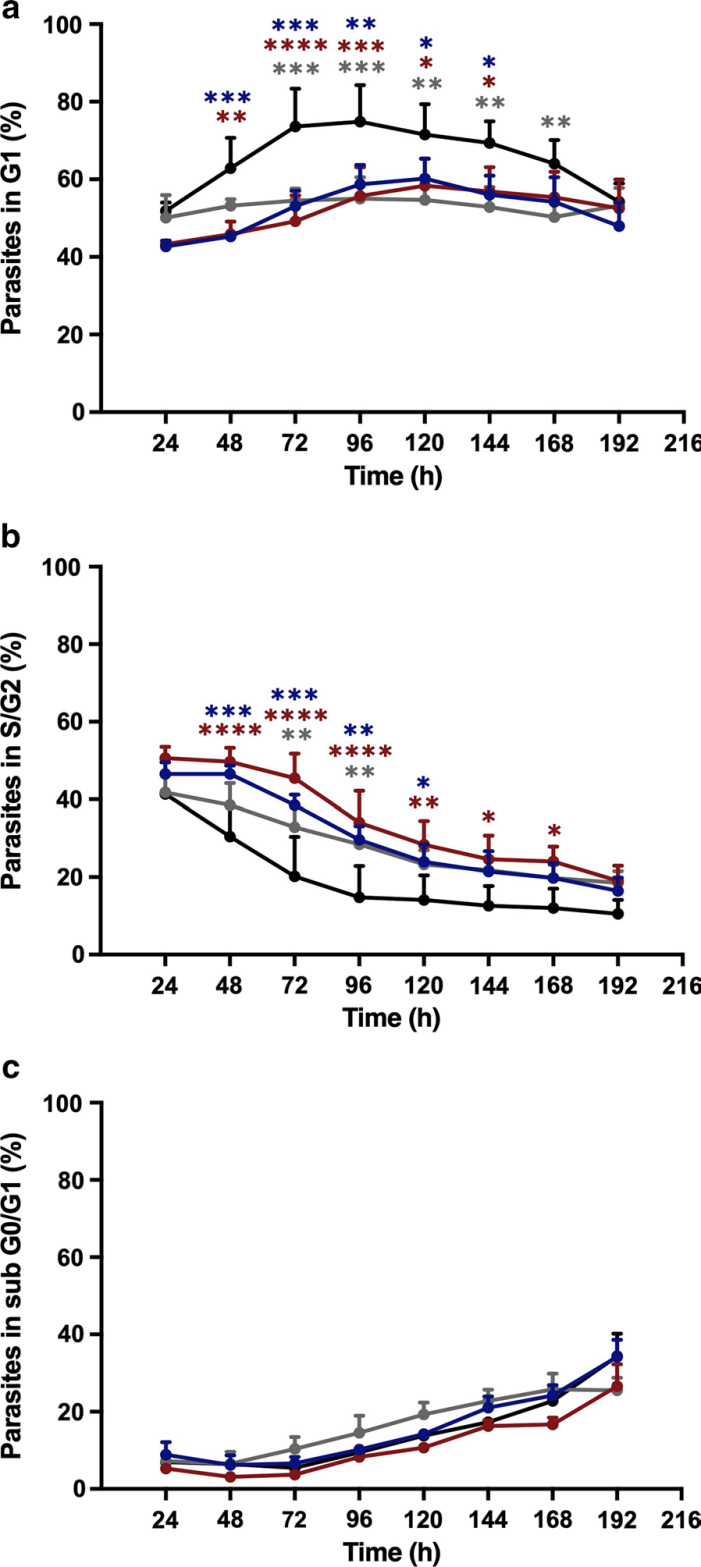
The cure-derived isolate revealed cell cycle arrest. The percentages of parasites in the G1 (**a**), S/G2 (**b**), and subG0/G1 (**c**) cell cycle phases are shown. Control isolate curves (IOC-L2335C) are shown in black. Failure-derived samples (IOC-L2371F) are displayed in gray. Resistant isolates selected in vitro (IOC-L2335R) are presented in dark blue in the absence of Sb(III) and in dark red when the media contained 8 µM Sb(III). Three biological replicates were assayed. Dots indicate means, and bars indicate the standard errors. Two-way ANOVA was used followed by Tukey’s HSD post hoc tests for IOC-L2335C. *P < 0.05, **P < 0.01, ***P < 0.001, and ****P < 0.0001

The amounts of DNA in different stages of the parasite’s life cycle were measured by flow cytometry with propidium iodide staining for cell cycle phase assessment [55]. A graphical representative analysis of the growth curve on the fifth day (stationary phase) is shown in Fig. 5. The values for all time courses are compared in Fig. 6. IOC-L2335C presented the highest percentage of cells in G1 (Fig. 6a), especially during the stationary phase (after the third day of the growth curve). Comparatively, this strain exhibited the lowest number of cells in S/G2 (Fig. 6b).

**Fig. 7:**
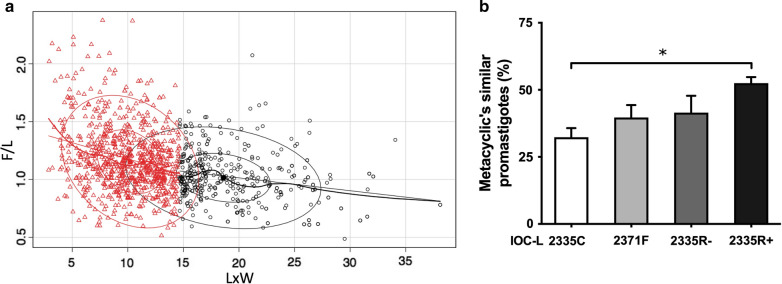
A higher proportion of metacyclic-like forms was found for the failure isolate and in vitro-selected resistant strain maintained with Sb(III). A graph of the body area (L × W) versus the flagellum-to-body length ratio (F/L) is presented, where **a** black circles represent predicted procyclic promastigotes, and red triangles represent predicted metacyclic-like promastigotes. The percentage of predicted metacyclic-like parasites for each isolate is shown (**b**). The IOC-L numbers of each isolate are indicated in the figure. The control (IOC-L2335C) is shown in white. The failure-derived sample (IOC-L2371F) is displayed in light gray. The resistant isolate selected in vitro (IOC-L2335R) is presented in dark gray in the absence of Sb(III) and in black when the media contained 8 μM Sb(III). Three biological replicates were assayed. The means and standard deviation among them are plotted. ANOVA was used, followed by Tukey’s HSD post hoc test. *P < 0.05

The morphology was determined by optical microscopy through culture slides. Two different measurements were obtained: the body area (L × W) and the ratio between the flagellum and body length (F/L). Procyclic forms were identified by higher L × W values and lower F/L ratios. Metacyclic-like forms were assigned to parasites with lower L × W values (< calculated average for each assay) and higher F/L ratios (F > L) (Fig. 7a). IOC-L2371F, 2335R-Sb(III), and 2335R + Sb(III) exhibited the higher proportions of metacyclic-like promastigotes compared to the strain IOC-L2335C (Fig. 7b). However, only the comparison with the lab-selected resistant strain under Sb(III) presented a significant difference.

### The maximum number of cells was affected by media sharing in co-culture assays

**Fig. 8:**
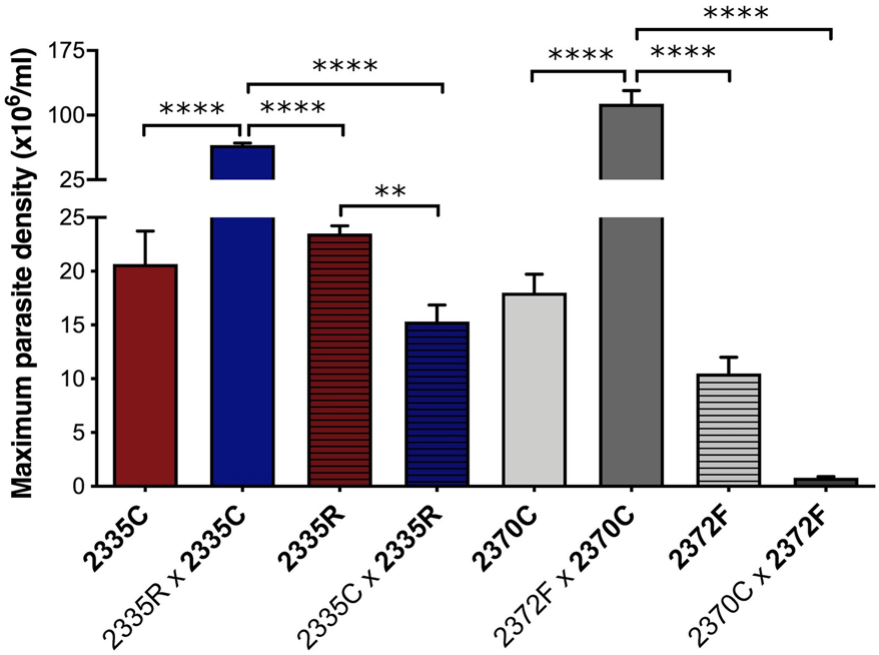
The maximum numbers of cells were affected by media sharing. Pairs are indicated in the graph, and the counted strain for each combination is shown in bold. Three biological replicates were assayed. The means and standard deviation among them are plotted. One-way ANOVA with Tukey’s HSD post hoc test was used. **P < 0.01 and ****P < 0.0001 for pairwise comparison

Co-culture assays were performed with two different combinations of strains sharing the same media in different compartments that were physically separated by a barrier within a Transwell system. Pairing was performed by combining more sensitive strains (IOC-L2335C or 2370C) with less sensitive strains (the lab-selected resistant strain, IOC-L2335R, or the strain from a treatment failure patient, IOC-L2372F) in the presence or absence of the drug (Fig. 8, see Additional file 1: Table S1 and Additional file 7: Table S6). For all combinations, Sb(III) eliminated co-cultured sensitive parasites. However, in the absence of Sb(III), IOC-L2335C and IOC-L2370C, the most susceptible strains, presented higher cell counts when co-cultured with less susceptible samples than the controls in a single culture, improving their growth capacity. Moreover, the opposite was observed for the less sensitive strains, which showed reduced cell densities when paired with the more sensitive ones. The IOC-L2335C single-culture control (dark red) showed an increase in the maximum density when co-cultured with IOC-L2335R (dark blue). In addition, the IOC-L2370C single-culture control (light gray) showed an increase in the maximum parasite density when co-cultured with IOC-L2372F (dark gray). Moreover, the presence of less susceptible samples (IOC-L2335R and IOC-L2372F) decreased the cell concentrations during co-culture (hachured dark red and dark gray for single cultures and hachured dark blue and gray for the co-cultures). IOC-L2335R significantly reduced maximum density when co-cultured with IOC-L2335C. Similarly, IOC-L2372F reached lower density when paired with IOC-L2370C, compared to the control.

After co-culture for 3 days, the Sb(III) IC_50_ was assayed and is shown in Table 1. A significant decrease in Sb(III) susceptibility was observed for IOC-L2370C after co-culture with IOC-L2372F compared to the control. However, the maintenance of IOC-L2335C in co-culture with IOC-L2335R did not affect the Sb(III) susceptibility.

**Table 1:** Enhancement of the Sb(III) IC_50_ for the less sensitive strain after growth under media sharing

Co-cultured strain	Tested strain	IC_50_ (µM)^a^	Previous IC_50_ (µM)^a^	Difference from the control (%) (*P-*value)
IOC-L2335R	IOC-L2335C	18.81 ± 4.81	24.20 ± 6.66	− 22.31 (*P* = *0.24*)
IOC-L2372F	IOC-L2370C	113.67 ± 3.56	35.36 ± 5.08	221.46 (*P* < *0.0001*)

^a^IC_50_ for Sb(III) of the tested strains (IOC-L 2335C and 2370C). Three biological replicates were assayed, and the means with the standard deviations are shown. A *t-*test was performed for paired comparisons, and the *P*-values are shown between parentheses

## Discussion

Treatment failure is multifactorial and influenced by several drug, patient and parasite features. Drug resistance represents one variable that might influence the final outcome. Thus, studies on the relationship between treatment failure and drug sensitivity within *Viannia* species are necessary. In the present study, *L.* (*V.*) *guyanensis* strains isolated before treatment from cured patients and patients with treatment failure were tested to determine different in vitro parameters. Additionally, one in vitro less sensitive lab-selected resistant strain was included. Analyses were conducted under drug pressure and/or in co-culture. The IC_50_ for Sb(III), in vitro growth patterns, metacyclic-like population proportion, and cell cycle dynamics were evaluated. All six tested samples revealed a correlation between treatment outcome and drug sensitivity. Although few samples were analyzed in the present study, the results are in agreement with previous observations that a good correlation was found between the recorded treatment outcome and Sb sensitivity for *L.* (*V.*) *guyanensis* and *L.* (*V.*) *braziliensis* [40, 56]. Conversely, a study by Yardley et al. [36] showed no correlation between treatment failure and Sb sensitivity for either parasite form in strains derived from patients from Peru [36].

All samples derived from failure cases exhibited a statistically higher IC_50_ for Sb(III) and showed higher in vitro growth mainly when subjected to drug pressure imposed by sublethal doses of Sb(III). These parameters suggest increased in vitro growth ability for the samples derived from failure cases. Indeed, the emergence of resistance in a population imposes an adaptive cost [57, 58]. However, in the specific context of *L.* (*L.*) *donovani* from Nepal, both field- and laboratory-derived resistant strains exhibited increased in vivo and in vitro fitness. Many explanations arise from this scenario, but the major hypotheses are as follows: (i) drug-selected parasites preserve or improve metabolic adaptations involving molecular pathways, leading not only to decreased antimony sensitivity but also to superior in vitro survival (e.g., increased thiol production flux) and persistence (according to the epi-phenotype hypothesis) [14], which is also supported by the evidence of cross-resistance between antimony and nitric oxide [43, 59]; (ii) features resulting in less sensitive subpopulations are already present in the initial polyclonal population, and the fittest strain emerged from this scenario [54]. Resistance to Sb(V) in *L. Viannia* populations not previously exposed to the drug suggests resistance emergence from a diverse response. Environmentally driven aneuploidy, recombination, and the high genetic heterogeneity of *L. Viannia* species populations support this assumption [60–62]. The Amazon forest region, where *L.* (*V.*) *guyanensis* is highly prevalent, has a very diverse environment. Vectors from different species are accountable for transmission [63, 64], and leishmanial species occur in sympatry, exhibiting high genetic heterogeneity [65–67]. These factors must have contributed even more to genetic and phenotypic variation and the emergence of diverse resistance mechanisms. Although the amastigote stage is exposed to the drug employed for leishmaniasis patient treatments, promastigotes are exposed to different compounds during phlebotomies blood and plant feeding, some with proved antileishmanial activity [68, 69]. Parasites usually develop innovative mechanisms to achieve drug resistance or other phenotypic traits. Furthermore, cross-resistance between drugs or these vector feeding-related compounds is feasible [70].

Cure- and failure-derived strains presented different growth patterns. The cure group exhibited more highly similar profiles, with an apparent reduction in the cell number under Sb(III) supplementation. The failure group showed less uniform profiles and an important reduction in the number of viable parasites under drug pressure during the first 48 h. Surprisingly, this was overcome by the emergence of a subpopulation presenting a different growth pattern. Most growth curves reflected a behavior resembling a hormetic phenomenon [71]. This result suggests that the initial polyclonal population resulting from failure-derived strains sheltered the subpopulation with increased Sb(III) resistance. Then, after sublethal drug pressure, selection could be conducted, leading to subclone emergence. Although Schneider’s media with undefined composition is not the ideal background to accompany these parasites' growth under drug challenge, all samples were exposed to the same conditions and batches. In a recent metabolomic study with *L. tropica*, parasites were cultivated with Schneider’s media to access biomarkers of parasitic antimony resistance [72]. It would be interesting to compare parasitic growth under defined or semi-defined media supplemented with known Sb(III) concentrations. However, it is challenging to isolate or cultivate most of *L.* (*Viannia*) parasites for few passages excluding fetal bovine serum (FBS), and several tests must be conducted.

Cell cycle measurement and morphometric analyses demonstrated that the cure-derived patient strain (IOC-L2335C) showed the highest proportion of cells in G1, the lowest numbers of cells in S/G2, and the lowest percentage of metacyclic-like cells compared to the strains derived from failure patients and the in vitro-selected strain less sensitive to Sb(III). This implies the arrest of replication and intense protein synthesis, with increased numbers of active metabolic cells. Accordingly, IOC-L2335C also displayed fewer metacyclic-like forms, which are known to be less proliferative. Finally, during synchronized in vitro growth, this strain produced fewer cells than the other strains, indicating its restricted growth capacity. These parameters could be associated with reduced infectivity. It is not known whether in vitro parasite growth is reflective of proliferation in phlebotomine species. However, if this is the case, a more highly proliferative strain could reach greater density and be favored during the transmission cycle [16]. A positive correlation between Sb(V) antimony resistance and metacyclogenesis was previously demonstrated for *L.* (*L.*) *donovani* [54, 61]. It was also observed for *L.* (*L.*) *donovani* that Sb(V)-resistant clinical strains revealed increased proteophosphoglycan expression (an important virulence factor), suggesting a relationship between drug sensitivity and infection capacity [17]. Thus, the present results with *L.* (*V.*) *guyanensis* led to a similar assumption, regardless of the differences between the drug presentation and phylogenetic distance between these two species (Additional file 4).

Co-culture improved the growth capacity of more sensitive samples, except during drug challenge, when it resulted in death even under co-culture. On the other hand, for the less sensitive strains, growth capacity was impaired by co-culture with more sensitive strains. Additionally, 3 days of media sharing increased the Sb(III) IC_50_ of the more sensitive strain. Co-culture thus decreased drug sensitivity. The conditions of preliminary co-culture before exposure to drug pressure could have allowed the emergence of a less sensitive population within the initial diverse polyclonal samples. Nevertheless, this condition was not present among cultures subjected to Sb(III) pressure from the beginning. Surprisingly, after media sharing, drug sensitivity reduction occurred only when the co-cultured pair comprised a failure-derived strain but not the less sensitive in vitro-selected strain. This corroborates data indicating that different mechanisms are associated with drug susceptibility within field-derived strains and laboratory-produced lineages [31].

These results suggest a role of intercellular communication, leading to growth ability and metabolic changes. Intercellular communication might be mediated by exosome vesicles or via the sharing of signaling molecules, as recently demonstrated for many unicellular organisms. Several examples of *quorum sensing* among bacterial pathogens have been described, resulting in differential responses during interactions with the environment and the host during invasion or differences in persistence capacity within a complex microbial community [73]. *Trypanosoma brucei* lacks G protein receptor signaling. Instead, an oligopeptidase transporter plays an important role in quorum sensing, as recently shown. Its activity and products represent a mechanism for paracrine signaling, leading to stumpy form development [74]. In an inbred mouse model, gut-commensal *Bacteroides* were able to control *Salmonella typhimurium* intestinal infection through short-chain fatty acid propionate secretion [75]. Communication through exosomes between parasites and host cells has been proposed and studied, including for leishmanial parasites. *Leishmania* spp. also secrete molecules via exosomes. These factors appear to contribute to pathogenesis by delivering protein virulence factors to macrophages [76]. During biting by the phlebotomine sand fly, exosomes and their components play a role along with metacyclic forms in vertebrate host infection [77]. *Leishmania* RNA virus 1 (LRV-1) is a leishmanial endosymbiont that leads to more severe disease development. It was recently demonstrated to be transmitted to uninfected parasites via exosomes [78]. Nevertheless, all samples in the present study lack LRV-1. Secretome analysis provided the first evidence of the role of these structures, such as the presence of GP63 (the major *Leishmania* spp. virulence factor), highlighting the ability of these structures to impair nitric oxide production by macrophages [79, 80]. Other in vitro and in vivo studies of infection in the presence of purified exosomes have demonstrated their immunomodulatory properties, leading to the impairment of TNF and IL-8 production and increased parasite load [77, 81]. Finally, *L.* (*L.*) *infantum* exosomes were differentially affected by drug exposure, affecting their abundance, characteristics (e.g. diameter), and cargo composition, including transcription and virulence factors and drug resistance-related targets [82].

Beyond the possible relationship between promastigote phenotypic characteristics and successful parasitic dispersion/infection, our results indicate the ability to share such characteristics without direct contact. Since it is not known how these promastigote characteristics could be expressed in amastigotes, the following observations must be interpreted with caution. Drug-driven selection may influence late patient refractoriness, which is often observed even after initial therapeutic success and results in reactivation or delayed clinical cure. Patients who are infected with strains less sensitive to antimony that are selected by the treatment could represent a source for the dispersion of these strains in the transmission cycle. These possibilities highlight the urgent need for studies about combined treatments. Most importantly, the early and assertive choice of an alternative drug for relapse treatment is important. It is important to keep in mind a feature observed for post kala-azar dermal leishmaniasis (PDKL), which is considered a complication of visceral leishmaniasis. The development of this disease form is highly related to irregular or inadequate treatments [83], where patients’ retreatment with different drugs is indicated [84].

## Conclusions

The data presented herein demonstrate that in vitro drug sensitivity was associated with treatment failure for the enrolled *L.* (*V.*) *guyanensis* strains. Moreover, reduced drug sensitivity was associated with a higher proportion of metacyclic-like forms and increased in vitro growth ability. Our results also indicate that intercellular communication can contribute to shaping parasite in vitro phenotypes, even without direct interaction between cells, through molecule secretion. Investigations of the mechanisms and precise functions underlying these observations for *Leishmania* spp. might contribute significantly to the understanding of parasite biology and leishmaniasis pathogenesis.

## Supplementary Information

**Additional file 1: Fig. S1** Growth capacity was incremented by media sharing. Curves for the cure-derived isolates co-cultured with the resistant in vitro-selected lineage (**a**) or failure-derived isolate (**b**) are shown. Black lines represent the more sensitive parasites cultured alone (C.T.): IOC-L2335C in a and 2370C in b. Light gray indicates the less sensitive IOC-L2335R cultured alone in a and 2372F in b, and dark red indicates C.T. cultured with 8 μM Sb(III). Dark gray represents more sensitive strains cultured with media shared from the less sensitive strains IOC-L2335C/2335R in a and IOC-L2370C/2372F in b; dark blue represents the same under 8 μM Sb(III). Three biological replicates were assayed. The means and standard deviation are plotted. Pairwise *t*-tests with adjustments of the confidence level by Sidak's method were performed, and the *P*-values for the pairwise comparisons at each time-point are listed in Additional file 5: Table S4 for graph a and Additional file 6: Table S5 for b, respectively]

**Additional file 2: Table S1.** Sample data.

**Additional file 3: Table S2.** Area under curve (AUC) values for Fig. 2 curves.

**Additional file 4: Table S3.** Sb(III)-induced resistance was persistent during passaging and cryopreservation.

**Additional file 5: Table S4.** *P*-values for pairwise comparisons among co-culture assays (resistant strain).

**Additional file 6: Table S5.** *P*-values for pairwise comparisons among co-culture assays (failure strain).

**Additional file 7: Table S6.** Co-culture strategies and density variations under media sharing.

## Data Availability

The datasets used and analyzed during the current study are available from the corresponding author on reasonable request.

## Abbreviations

FBS: Fetal bovine serum
IC_50_: Half maximal inhibitory concentration
NNN: Novy-MacNeal-Nicolle
NO: Nitric oxide
Sb(III): Trivalent antimonial
Sb(V): Sodium stibogluconate, pentavalent antimonial
