# Supplementary figures and images for: Insights from Leishmania (Viannia) guyanensis in vitro behavior and intercellular communication
Source: Parasit Vectors. 2021 Oct 28;14:556. doi: 10.1186/s13071-021-05057-x (PMC8554959; doi:10.1186/s13071-021-05057-x)

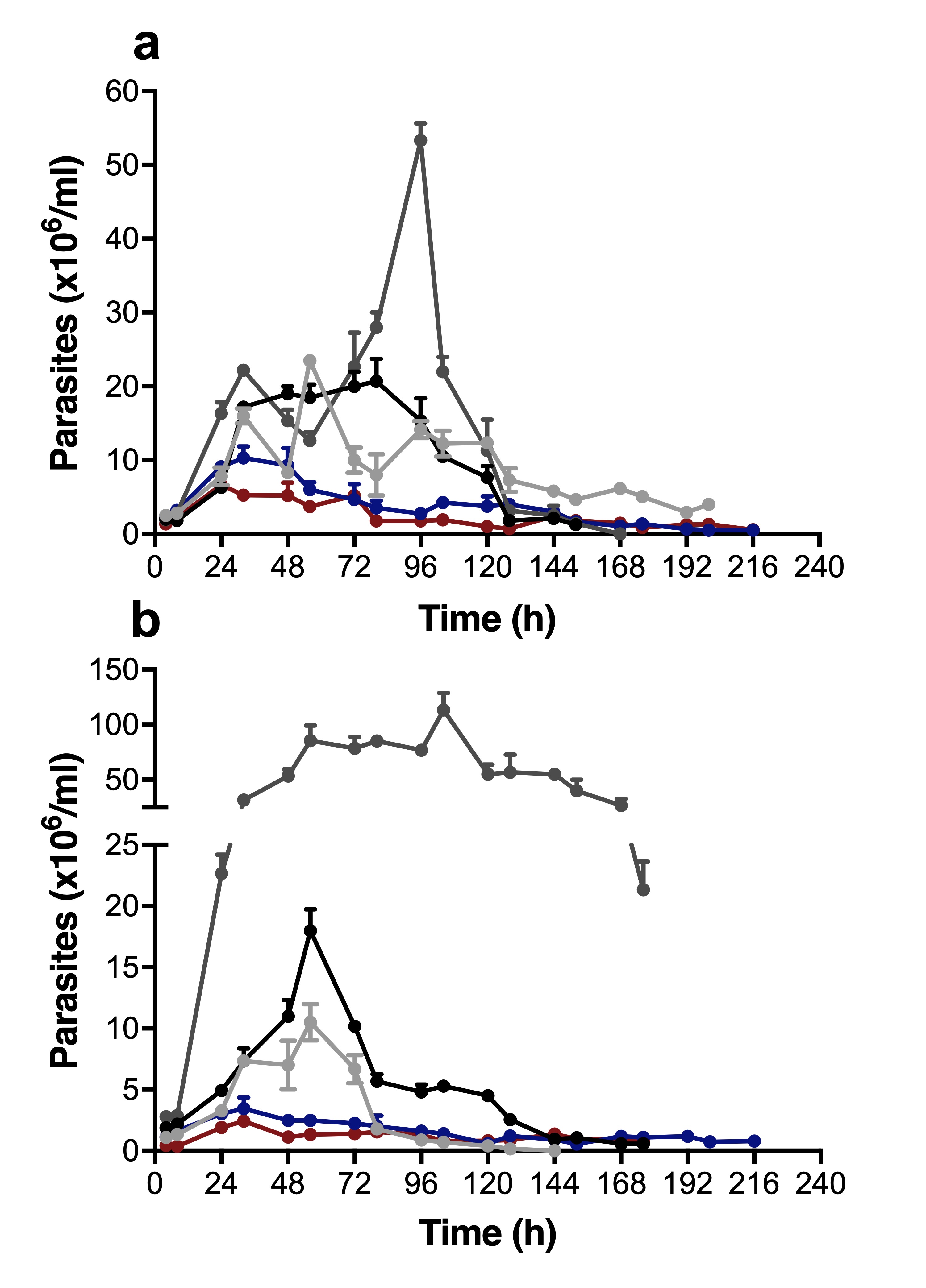

Supplement: Supplementary file 1 — Additional file 1: Fig. S1 Growth capacity was incremented by media sharing. Curves for the cure-derived isolates co-cultured with the resistant in vitro-selected lineage (a) or failure-derived isolate (b) are shown. Black lines represent the more sensitive parasites cultured alone (C.T.): IOC-L2335C in a and 2370C in b. Light gray indicates the less sensitive IOC-L2335R cultured alone in a and 2372F in b, and dark red indicates C.T. cultured with 8 μM Sb(III). Dark gray represents more sensitive strains cultured with media shared from the less sensitive strains IOC-L2335C/2335R in a and IOC-L2370C/2372F in b; dark blue represents the same under 8 μM Sb(III). Three biological replicates were assayed. The means and standard deviation are plotted. Pairwise t-tests with adjustments of the confidence level by Sidak's method were performed, and the P-values for the pairwise comparisons at each time-point are listed in Additional file 5: Table S4 for graph a and Additional file 6: Table S5 for b, respectively] [file 13071_2021_5057_MOESM1_ESM.jpg]
