# Supplementary material for: Insights from Leishmania (Viannia) guyanensis in vitro behavior and intercellular communication
Source: Parasit Vectors. 2021 Oct 28;14:556. doi: 10.1186/s13071-021-05057-x (PMC8554959; doi:10.1186/s13071-021-05057-x)
Supplement: Supplementary file 2 — Additional file 2: Table S1. Sample data. [file 13071_2021_5057_MOESM2_ESM.docx]

**Table S1:** Sample data.

| **IOC-L** | **International Code** | **Outcome*** | **Clinical presentation** | **Brazilian State of sample origin** |
| --- | --- | --- | --- | --- |
| **2335** | MHOM/BR/1997/NMT-MAO 203P | Cure | LC | AM |
| **2370** | MHOM/BR/1997/NMT-MAO 264P | Cure | LC | AM |
| **2960** | MHOM/BR/2007/029-ZAV | Cure | LC | AM |
| **2354** | MHOM/BR/1997/NMT-MAO 243P | Failure | LC | AM |
| **2371** | MHOM/BR/1997/NMT-MAO 292P | Failure | LC | AM |
| **2372** | MHOM/BR/1997/NMT-MAO 292G | Failure | LC | AM |

***** Patients subjected to 20 days of intravenous or intramuscular treatment with 20 mg/kg/day of meglumine antimoniate (Glucantime®, Rhodia, São Paulo, Brazil) were classified as cured when full recovery and lesion epithelization were reached within three months. If the disease persisted, representing treatment relapse or symptoms that had poorly evolved during the same period, the patients were diagnosed with therapeutic failure or relapse.
