# Supplementary material for: Insights from Leishmania (Viannia) guyanensis in vitro behavior and intercellular communication
Source: Parasit Vectors. 2021 Oct 28;14:556. doi: 10.1186/s13071-021-05057-x (PMC8554959; doi:10.1186/s13071-021-05057-x)
Supplement: Supplementary file 3 — Additional file 3: Table S2. Area under curve (AUC) values for Fig. 2 curves. [file 13071_2021_5057_MOESM3_ESM.docx]

**Table S2:** Area under curves (AUC) values for Figure 2 curves.

| **Sample** | **Curves** | **Mean** | **SE** | ***p*-value** |
| --- | --- | --- | --- | --- |
| **IOC-L2372** | -Sb(III) | 5.802276 | 0.007442 | 4.03173E-06 |
|  | +Sb(III) | 6.169392 | 0.007442 |  |
| **IOC-L2370** | -Sb(III) | 6.012788 | 0.005206 | 8.09462E-08 |
|  | +Sb(III) | 5.329785 | 0.005206 |  |
| **IOC-L2371** | -Sb(III) | 6.104204 | 0.017101 | 9.4026E-05 |
|  | +Sb(III) | 5.722369 | 0.017101 |  |
| **IOC-L2335** | -Sb(III) | 5.993107 | 0.010822 | 0.000556565 |
|  | +Sb(III) | 5.83968 | 0.010822 |  |
| **IOC-L2354** | -Sb(III) | 6.263589 | 0.012061 | 0.203301355 |
|  | +Sb(III) | 6.289504 | 0.012061 |  |
| **IOC-L2960** | -Sb(III) | 6.222815 | 0.022376 | 1.84829E-05 |
|  | +Sb(III) | 5.469683 | 0.022376 |  |

SE, standard error

*p*-values for area under curves comparison between untreated and Sb(III) treated samples
