# Supplementary material for: Insights from Leishmania (Viannia) guyanensis in vitro behavior and intercellular communication
Source: Parasit Vectors. 2021 Oct 28;14:556. doi: 10.1186/s13071-021-05057-x (PMC8554959; doi:10.1186/s13071-021-05057-x)
Supplement: Supplementary file 4 — Additional file 4: Table S3. Sb(III)-induced resistance was persistent during passaging and cryopreservation. [file 13071_2021_5057_MOESM4_ESM.docx]

**Table S3:** Sb(III)-induced resistance was persistent during passages and cryopreservation.

| **Strain** | **IC50 Sb(III)**  **(μM)** |
| --- | --- |
| IOC-L2335C | 24.20±6.66 |
| IOC-L2335R-Sb(III) | 83.81±2.09* |
| IOC-L2335R+Sb(III) | 96.42±11.19* |
| IOC-L2335R cryo+Sb(III) | 110.02±2.77* |
| IOC-L2335R cryo-Sb(III) | 107.41±12.99* |
| IOC-L2371F | 81.66±5.06* |

Cure (IOC-L2335C)- and failure (IOC-L2371F)-derived isolates as well as IOC-L2335R, whose resistance was selected, were maintained or cryopreserved (cryo) with (+Sb(III)) or without Sb(III) (-Sb(III)) and showed diverse IC50 values. Three biological replicates were assayed, and the mean and standard deviation are shown. Stars indicate a *p-*value *< 0.001* compared to IOC-L2335C using ANOVA followed by Dunnett's multiple comparison test.
