# Supplementary material for: Insights from Leishmania (Viannia) guyanensis in vitro behavior and intercellular communication
Source: Parasit Vectors. 2021 Oct 28;14:556. doi: 10.1186/s13071-021-05057-x (PMC8554959; doi:10.1186/s13071-021-05057-x)
Supplement: Supplementary file 5 — Additional file 5: Table S4. P-values for pairwise comparisons among co-culture assays (resistant strain). [file 13071_2021_5057_MOESM5_ESM.docx]

**Table S4:** *P*-values for pairwise comparisons among co-cultures with the *in vitro*-selected resistant isolate (Figure S1-A). *P*-values *< 0.05* are shown in bold.

| **Time (h)** | **Co-culture pair** | ***p*-value** |
| --- | --- | --- |
| **4** | IOC-L2335/2335+Sb(III) - IOC-L2335/2335(C.T.) | **0,009928723** |
|  | IOC-L2335/2335+Sb(III) - IOC-L2335/2335R-Sb(III) | 0,108893697 |
|  | IOC-L2335/2335+Sb(III) - IOC-L2335R/2335R | 3,01597E-05 |
|  | IOC-L2335/2335+Sb(III) - IOC-L2335/2335R+Sb(III) | **0,004872278** |
|  | IOC-L2335/2335(C.T.) - IOC-L2335/2335R-Sb(III) | 0,551744182 |
|  | IOC-L2335/2335(C.T.) - IOC-L2335R/2335R | **0,005784978** |
|  | IOC-L2335/2335(C.T.) - IOC-L2335/2335R+Sb(III) | 0,987272972 |
|  | IOC-L2335/2335R-Sb(III) - IOC-L2335R/2335R | **0,000685427** |
|  | IOC-L2335/2335R-Sb(III) - IOC-L2335/2335R+Sb(III) | 0,314395714 |
|  | IOC-L2335R/2335R - IOC-L2335/2335R+Sb(III) | **0,011858797** |
| **8** | IOC-L2335/2335+Sb(III) - IOC-L2335/2335(C.T.) | 0,275818333 |
|  | IOC-L2335/2335+Sb(III) - IOC-L2335/2335R-Sb(III) | 0,729855491 |
|  | IOC-L2335/2335+Sb(III) - IOC-L2335R/2335R | 0,231837507 |
|  | IOC-L2335/2335+Sb(III) - IOC-L2335/2335R+Sb(III) | 0,038847857 |
|  | IOC-L2335/2335(C.T.) - IOC-L2335/2335R-Sb(III) | **0,04346882** |
|  | IOC-L2335/2335(C.T.) - IOC-L2335R/2335R | **0,009016055** |
|  | IOC-L2335/2335(C.T.) - IOC-L2335/2335R+Sb(III) | **0,001660123** |
|  | IOC-L2335/2335R-Sb(III) - IOC-L2335R/2335R | 0,837627664 |
|  | IOC-L2335/2335R-Sb(III) - IOC-L2335/2335R+Sb(III) | 0,250031454 |
|  | IOC-L2335R/2335R - IOC-L2335/2335R+Sb(III) | 0,759119011 |
| **24** | IOC-L2335/2335+Sb(III) - IOC-L2335/2335(C.T.) | 0,751586157 |
|  | IOC-L2335/2335+Sb(III) - IOC-L2335/2335R-Sb(III) | 5,08051E-06 |
|  | IOC-L2335/2335+Sb(III) - IOC-L2335R/2335R | 0,795267866 |
|  | IOC-L2335/2335+Sb(III) - IOC-L2335/2335R+Sb(III) | 0,0645288 |
|  | IOC-L2335/2335(C.T.) - IOC-L2335/2335R-Sb(III) | **1,92982E-06** |
|  | IOC-L2335/2335(C.T.) - IOC-L2335R/2335R | 0,216724632 |
|  | IOC-L2335/2335(C.T.) - IOC-L2335/2335R+Sb(III) | **0,010183881** |
|  | IOC-L2335/2335R-Sb(III) - IOC-L2335R/2335R | **1,35491E-05** |
|  | IOC-L2335/2335R-Sb(III) - IOC-L2335/2335R+Sb(III) | **0,000101701** |
|  | IOC-L2335R/2335R - IOC-L2335/2335R+Sb(III) | 0,327011164 |
| **32** | IOC-L2335/2335+Sb(III) - IOC-L2335/2335(C.T.) | **6,18235E-07** |
|  | IOC-L2335/2335+Sb(III) - IOC-L2335/2335R-Sb(III) | 2,04668E-08 |
|  | IOC-L2335/2335+Sb(III) - IOC-L2335R/2335R | 7,02792E-07 |
|  | IOC-L2335/2335+Sb(III) - IOC-L2335/2335R+Sb(III) | **0,000525287** |
|  | IOC-L2335/2335(C.T.) - IOC-L2335/2335R-Sb(III) | **0,001089471** |
|  | IOC-L2335/2335(C.T.) - IOC-L2335R/2335R | 0,999680592 |
|  | IOC-L2335/2335(C.T.) - IOC-L2335/2335R+Sb(III) | **0,000196941** |
|  | IOC-L2335/2335R-Sb(III) - IOC-L2335R/2335R | **0,000854354** |
|  | IOC-L2335/2335R-Sb(III) - IOC-L2335/2335R+Sb(III) | 9,20365E-07 |
|  | IOC-L2335R/2335R - IOC-L2335/2335R+Sb(III) | **0,000244697** |
| **48** | IOC-L2335/2335+Sb(III) - IOC-L2335/2335(C.T.) | **3,2653E-06** |
|  | IOC-L2335/2335+Sb(III) - IOC-L2335/2335R-Sb(III) | **1,99617E-05** |
|  | IOC-L2335/2335+Sb(III) - IOC-L2335R/2335R | 0,066606594 |
|  | IOC-L2335/2335+Sb(III) - IOC-L2335/2335R+Sb(III) | **0,002434468** |
|  | IOC-L2335/2335(C.T.) - IOC-L2335/2335R-Sb(III) | 0,304703123 |
|  | IOC-L2335/2335(C.T.) - IOC-L2335R/2335R | **5,61959E-05** |
|  | IOC-L2335/2335(C.T.) - IOC-L2335/2335R+Sb(III) | **0,000720018** |
|  | IOC-L2335/2335R-Sb(III) - IOC-L2335R/2335R | **0,000599815** |
|  | IOC-L2335/2335R-Sb(III) - IOC-L2335/2335R+Sb(III) | **0,013036977** |
|  | IOC-L2335R/2335R - IOC-L2335/2335R+Sb(III) | 0,251266222 |
| **56** | IOC-L2335/2335+Sb(III) - IOC-L2335/2335(C.T.) | **1,55291E-08** |
|  | IOC-L2335/2335+Sb(III) - IOC-L2335/2335R-Sb(III) | **1,09229E-06** |
|  | IOC-L2335/2335+Sb(III) - IOC-L2335R/2335R | **3,83352E-10** |
|  | IOC-L2335/2335+Sb(III) - IOC-L2335/2335R+Sb(III) | 0,071256156 |
|  | IOC-L2335/2335(C.T.) - IOC-L2335/2335R-Sb(III) | **0,000217257** |
|  | IOC-L2335/2335(C.T.) - IOC-L2335R/2335R | **8,86682E-05** |
|  | IOC-L2335/2335(C.T.) - IOC-L2335/2335R+Sb(III) | **8,30184E-08** |
|  | IOC-L2335/2335R-Sb(III) - IOC-L2335R/2335R | **2,44331E-07** |
|  | IOC-L2335/2335R-Sb(III) - IOC-L2335/2335R+Sb(III) | **1,34056E-05** |
|  | IOC-L2335R/2335R - IOC-L2335/2335R+Sb(III) | **1,06567E-09** |
| **72** | IOC-L2335/2335+Sb(III) - IOC-L2335/2335(C.T.) | **0,000823705** |
|  | IOC-L2335/2335+Sb(III) - IOC-L2335/2335R-Sb(III) | **4,85738E-05** |
|  | IOC-L2335/2335+Sb(III) - IOC-L2335R/2335R | 0,209494873 |
|  | IOC-L2335/2335+Sb(III) - IOC-L2335/2335R+Sb(III) | 0,996907479 |
|  | IOC-L2335/2335(C.T.) - IOC-L2335/2335R-Sb(III) | 0,17769338 |
|  | IOC-L2335/2335(C.T.) - IOC-L2335R/2335R | **0,023291183** |
|  | IOC-L2335/2335(C.T.) - IOC-L2335/2335R+Sb(III) | **0,00053984** |
|  | IOC-L2335/2335R-Sb(III) - IOC-L2335R/2335R | **0,00071058** |
|  | IOC-L2335/2335R-Sb(III) - IOC-L2335/2335R+Sb(III) | **3,46862E-05** |
|  | IOC-L2335R/2335R - IOC-L2335/2335R+Sb(III) | 0,129014763 |
| **80** | IOC-L2335/2335+Sb(III) - IOC-L2335/2335(C.T.) | **2,14238E-06** |
|  | IOC-L2335/2335+Sb(III) - IOC-L2335/2335R-Sb(III) | **5,95481E-08** |
|  | IOC-L2335/2335+Sb(III) - IOC-L2335R/2335R | **0,000623505** |
|  | IOC-L2335/2335+Sb(III) - IOC-L2335/2335R+Sb(III) | 0,823186572 |
|  | IOC-L2335/2335(C.T.) - IOC-L2335/2335R-Sb(III) | **0,001535131** |
|  | IOC-L2335/2335(C.T.) - IOC-L2335R/2335R | **0,001377912** |
|  | IOC-L2335/2335(C.T.) - IOC-L2335/2335R+Sb(III) | **5,04835E-06** |
|  | IOC-L2335/2335R-Sb(III) - IOC-L2335R/2335R | **3,71781E-06** |
|  | IOC-L2335/2335R-Sb(III) - IOC-L2335/2335R+Sb(III) | **1,06092E-07** |
|  | IOC-L2335R/2335R - IOC-L2335/2335R+Sb(III) | **0,002544086** |
| **96** | IOC-L2335/2335+Sb(III) - IOC-L2335/2335(C.T.) | **0,000844816** |
|  | IOC-L2335/2335+Sb(III) - IOC-L2335/2335R-Sb(III) | **4,05884E-10** |
|  | IOC-L2335/2335+Sb(III) - IOC-L2335R/2335R | **0,000298158** |
|  | IOC-L2335/2335+Sb(III) - IOC-L2335/2335R+Sb(III) | 0,999969728 |
|  | IOC-L2335/2335(C.T.) - IOC-L2335/2335R-Sb(III) | **6,03367E-09** |
|  | IOC-L2335/2335(C.T.) - IOC-L2335R/2335R | 0,912743032 |
|  | IOC-L2335/2335(C.T.) - IOC-L2335/2335R+Sb(III) | **0,000965692** |
|  | IOC-L2335/2335R-Sb(III) - IOC-L2335R/2335R | **9,72135E-09** |
|  | IOC-L2335/2335R-Sb(III) - IOC-L2335/2335R+Sb(III) | **4,16444E-10** |
|  | IOC-L2335R/2335R - IOC-L2335/2335R+Sb(III) | **0,000337373** |
| **104** | IOC-L2335/2335+Sb(III) - IOC-L2335/2335(C.T.) | **7,21386E-07** |
|  | IOC-L2335/2335+Sb(III) - IOC-L2335/2335R-Sb(III) | **1,0876E-10** |
|  | IOC-L2335/2335+Sb(III) - IOC-L2335R/2335R | **6,93252E-08** |
|  | IOC-L2335/2335+Sb(III) - IOC-L2335/2335R+Sb(III) | **0,010686016** |
|  | IOC-L2335/2335(C.T.) - IOC-L2335/2335R-Sb(III) | **7,43953E-09** |
|  | IOC-L2335/2335(C.T.) - IOC-L2335R/2335R | **0,019573387** |
|  | IOC-L2335/2335(C.T.) - IOC-L2335/2335R+Sb(III) | **2,47553E-05** |
|  | IOC-L2335/2335R-Sb(III) - IOC-L2335R/2335R | **6,44746E-08** |
|  | IOC-L2335/2335R-Sb(III) - IOC-L2335/2335R+Sb(III) | **2,31965E-10** |
|  | IOC-L2335R/2335R - IOC-L2335/2335R+Sb(III) | **9,55952E-07** |
| **120** | IOC-L2335/2335+Sb(III) - IOC-L2335/2335(C.T.) | **0,042399812** |
|  | IOC-L2335/2335+Sb(III) - IOC-L2335/2335R-Sb(III) | **0,000291643** |
|  | IOC-L2335/2335+Sb(III) - IOC-L2335R/2335R | **0,001448505** |
|  | IOC-L2335/2335+Sb(III) - IOC-L2335/2335R+Sb(III) | 0,525227806 |
|  | IOC-L2335/2335(C.T.) - IOC-L2335/2335R-Sb(III) | **0,032786102** |
|  | IOC-L2335/2335(C.T.) - IOC-L2335R/2335R | 0,222247492 |
|  | IOC-L2335/2335(C.T.) - IOC-L2335/2335R+Sb(III) | 0,428542127 |
|  | IOC-L2335/2335R-Sb(III) - IOC-L2335R/2335R | 0,716610014 |
|  | IOC-L2335/2335R-Sb(III) - IOC-L2335/2335R+Sb(III) | **0,002308612** |
|  | IOC-L2335R/2335R - IOC-L2335/2335R+Sb(III) | **0,014600513** |
| **128** | IOC-L2335/2335+Sb(III) - IOC-L2335/2335(C.T.) | 0,997564447 |
|  | IOC-L2335/2335+Sb(III) - IOC-L2335/2335R-Sb(III) | 0,348019783 |
|  | IOC-L2335/2335+Sb(III) - IOC-L2335R/2335R | **0,016703997** |
|  | IOC-L2335/2335+Sb(III) - IOC-L2335/2335R+Sb(III) | 0,069044362 |
|  | IOC-L2335/2335(C.T.) - IOC-L2335/2335R-Sb(III) | 0,501956689 |
|  | IOC-L2335/2335(C.T.) - IOC-L2335R/2335R | **0,027020878** |
|  | IOC-L2335/2335(C.T.) - IOC-L2335/2335R+Sb(III) | 0,111367919 |
|  | IOC-L2335/2335R-Sb(III) - IOC-L2335R/2335R | 0,315686098 |
|  | IOC-L2335/2335R-Sb(III) - IOC-L2335/2335R+Sb(III) | 0,79269504 |
|  | IOC-L2335R/2335R - IOC-L2335/2335R+Sb(III) | 0,883820222 |
| **144** | IOC-L2335/2335+Sb(III) - IOC-L2335/2335(C.T.) | 0,653408963 |
|  | IOC-L2335/2335+Sb(III) - IOC-L2335/2335R-Sb(III) | 0,990267767 |
|  | IOC-L2335/2335+Sb(III) - IOC-L2335R/2335R | 7,57513E-07 |
|  | IOC-L2335/2335+Sb(III) - IOC-L2335/2335R+Sb(III) | **0,009413183** |
|  | IOC-L2335/2335(C.T.) - IOC-L2335/2335R-Sb(III) | 0,410336785 |
|  | IOC-L2335/2335(C.T.) - IOC-L2335R/2335R | 3,04799E-07 |
|  | IOC-L2335/2335(C.T.) - IOC-L2335/2335R+Sb(III) | **0,001332363** |
|  | IOC-L2335/2335R-Sb(III) - IOC-L2335R/2335R | **1,04354E-06** |
|  | IOC-L2335/2335R-Sb(III) - IOC-L2335/2335R+Sb(III) | **0,018601324** |
|  | IOC-L2335R/2335R - IOC-L2335/2335R+Sb(III) | **2,87831E-05** |
| **152** | IOC-L2335/2335+Sb(III) - IOC-L2335/2335(C.T.) | 0,052551299 |
|  | IOC-L2335/2335+Sb(III) - IOC-L2335/2335R-Sb(III) | **0,039251293** |
|  | IOC-L2335/2335+Sb(III) - IOC-L2335R/2335R | **9,58681E-07** |
|  | IOC-L2335/2335+Sb(III) - IOC-L2335/2335R+Sb(III) | 0,171387174 |
|  | IOC-L2335/2335(C.T.) - IOC-L2335/2335R-Sb(III) | 0,999668653 |
|  | IOC-L2335/2335(C.T.) - IOC-L2335R/2335R | **1,2274E-07** |
|  | IOC-L2335/2335(C.T.) - IOC-L2335/2335R+Sb(III) | 0,930994304 |
|  | IOC-L2335/2335R-Sb(III) - IOC-L2335R/2335R | **1,1054E-07** |
|  | IOC-L2335/2335R-Sb(III) - IOC-L2335/2335R+Sb(III) | 0,863779844 |
|  | IOC-L2335R/2335R - IOC-L2335/2335R+Sb(III) | **1,91602E-07** |
