# Supplementary material for: Insights from Leishmania (Viannia) guyanensis in vitro behavior and intercellular communication
Source: Parasit Vectors. 2021 Oct 28;14:556. doi: 10.1186/s13071-021-05057-x (PMC8554959; doi:10.1186/s13071-021-05057-x)
Supplement: Supplementary file 6 — Additional file 6: Table S5. P-values for pairwise comparisons among co-culture assays (failure strain). [file 13071_2021_5057_MOESM6_ESM.docx]

**Table S5:** *P*-values for pairwise comparisons of co-cultures with the failure-derived isolate (Figure S1-B). *P*-values *< 0.05* are shown in bold.

| **Time (h)** | **Co-culture pair** | ***p*-value** |
| --- | --- | --- |
| **4** | IOC-L2370/2370+Sb(III) - IOC-L2370/2372+Sb(III) | **8,71216E-05** |
|  | IOC-L2370/2370+Sb(III) - IOC-L2370/2372-Sb(III) | **4,83587E-10** |
|  | IOC-L2370/70+Sb(III) - IOC-L2370/2370(C.T.) | **1,05637E-07** |
|  | IOC-L2370/2370+Sb(III) - IOC-L2372/2372 | **0,000774597** |
|  | IOC-L2370/72+Sb(III) - IOC-L2370/2372-Sb(III) | **2,5792E-08** |
|  | IOC-L2370/2372+Sb(III) - IOC-L2370/2370(C.T.) | **4,50797E-05** |
|  | IOC-L2370/2372+Sb(III) - IOC-L2372/2372 | 0,398127362 |
|  | IOC-L2370/2372-Sb(III) - IOC-L2370/2370(C.T.) | **7,13127E-06** |
|  | IOC-L2370/2372-Sb(III) - IOC-L2372/2372 | **9,13211E-09** |
|  | IOC-L2370/2370(C.T.) - IOC-L2372/2372 | **7,83184E-06** |
| **8** | IOC-L2370/2370+Sb(III) - IOC-L2370/2372+Sb(III) | **5,7016E-05** |
|  | IOC-L2370/2370+Sb(III) - IOC-L2370/2372-Sb(III) | **1,41211E-07** |
|  | IOC-L2370/70+Sb(III) - IOC-L2370/2370(C.T.) | **1,91439E-06** |
|  | IOC-L2370/2370+Sb(III) - IOC-L2372/2372 | **0,000359977** |
|  | IOC-L2370/72+Sb(III) - IOC-L2370/2372-Sb(III) | **0,000119837** |
|  | IOC-L2370/2372+Sb(III) - IOC-L2370/2370(C.T.) | **0,022883085** |
|  | IOC-L2370/2372+Sb(III) - IOC-L2372/2372 | 0,50779375 |
|  | IOC-L2370/2372-Sb(III) - IOC-L2370/2370(C.T.) | **0,018381195** |
|  | IOC-L2370/2372-Sb(III) - IOC-L2372/2372 | **2,20997E-05** |
|  | IOC-L2370/2370(C.T.) - IOC-L2372/2372 | **0,002065873** |
| **24** | IOC-L2370/2370+Sb(III) - IOC-L2370/2372+Sb(III) | 0,067488075 |
|  | IOC-L2370/2370+Sb(III) - IOC-L2370/2372-Sb(III) | **3,83693E-13** |
|  | IOC-L2370/70+Sb(III) - IOC-L2370/2370(C.T.) | **0,000290305** |
|  | IOC-L2370/2370+Sb(III) - IOC-L2372/2372 | **0,04172095** |
|  | IOC-L2370/72+Sb(III) - IOC-L2370/2372-Sb(III) | **9,49241E-13** |
|  | IOC-L2370/2372+Sb(III) - IOC-L2370/2370(C.T.) | **0,020506092** |
|  | IOC-L2370/2372+Sb(III) - IOC-L2372/2372 | 0,997635033 |
|  | IOC-L2370/2372-Sb(III) - IOC-L2370/2370(C.T.) | **2,84706E-12** |
|  | IOC-L2370/2372-Sb(III) - IOC-L2372/2372 | **1,03872E-12** |
|  | IOC-L2370/2370(C.T.) - IOC-L2372/2372 | **0,033120215** |
| **32** | IOC-L2370/2370+Sb(III) - IOC-L2370/2372+Sb(III) | **0,006908777** |
|  | IOC-L2370/2370+Sb(III) - IOC-L2370/2372-Sb(III) | **3,61933E-14** |
|  | IOC-L2370/70+Sb(III) - IOC-L2370/2370(C.T.) | **3,37793E-06** |
|  | IOC-L2370/2370+Sb(III) - IOC-L2372/2372 | **8,73372E-06** |
|  | IOC-L2370/72+Sb(III) - IOC-L2370/2372-Sb(III) | **5,70655E-14** |
|  | IOC-L2370/2372+Sb(III) - IOC-L2370/2370(C.T.) | **0,000308033** |
|  | IOC-L2370/2372+Sb(III) - IOC-L2372/2372 | **0,001271004** |
|  | IOC-L2370/2372-Sb(III) - IOC-L2370/2370(C.T.) | **2,99094E-13** |
|  | IOC-L2370/2372-Sb(III) - IOC-L2372/2372 | **2,17382E-13** |
|  | IOC-L2370/2370(C.T.) - IOC-L2372/2372 | 0,79227344 |
| **48** | IOC-L2370/2370+Sb(III) - IOC-L2370/2372+Sb(III) | 0,949082505 |
|  | IOC-L2370/2370+Sb(III) - IOC-L2370/2372-Sb(III) | **8,83035E-10** |
|  | IOC-L2370/70+Sb(III) - IOC-L2370/2370(C.T.) | **0,009184954** |
|  | IOC-L2370/2370+Sb(III) - IOC-L2372/2372 | 0,120586443 |
|  | IOC-L2370/72+Sb(III) - IOC-L2370/2372-Sb(III) | **1,20914E-09** |
|  | IOC-L2370/2372+Sb(III) - IOC-L2370/2370(C.T.) | **0,027174572** |
|  | IOC-L2370/2372+Sb(III) - IOC-L2372/2372 | 0,329190292 |
|  | IOC-L2370/2372-Sb(III) - IOC-L2370/2370(C.T.) | **8,89525E-09** |
|  | IOC-L2370/2372-Sb(III) - IOC-L2372/2372 | **3,43458E-09** |
|  | IOC-L2370/2370(C.T.) - IOC-L2372/2372 | 0,48653247 |
| **56** | IOC-L2370/2370+Sb(III) - IOC-L2370/2372+Sb(III) | 0,994606479 |
|  | IOC-L2370/2370+Sb(III) - IOC-L2370/2372-Sb(III) | **1,46252E-10** |
|  | IOC-L2370/70+Sb(III) - IOC-L2370/2370(C.T.) | **0,00014744** |
|  | IOC-L2370/2370+Sb(III) - IOC-L2372/2372 | **0,017586854** |
|  | IOC-L2370/72+Sb(III) - IOC-L2370/2372-Sb(III) | **1,56467E-10** |
|  | IOC-L2370/2372+Sb(III) - IOC-L2370/2370(C.T.) | **0,000229334** |
|  | IOC-L2370/2372+Sb(III) - IOC-L2372/2372 | **0,031814613** |
|  | IOC-L2370/2372-Sb(III) - IOC-L2370/2370(C.T.) | **7,89042E-10** |
|  | IOC-L2370/2372-Sb(III) - IOC-L2372/2372 | **2,95034E-10** |
|  | IOC-L2370/2370(C.T.) - IOC-L2372/2372 | **0,031863274** |
| **72** | IOC-L2370/2370+Sb(III) - IOC-L2370/2372+Sb(III) | 0,99866057 |
|  | IOC-L2370/2370+Sb(III) - IOC-L2370/2372-Sb(III) | **4,96651E-09** |
|  | IOC-L2370/70+Sb(III) - IOC-L2370/2370(C.T.) | 0,098308673 |
|  | IOC-L2370/2370+Sb(III) - IOC-L2372/2372 | 0,492388388 |
|  | IOC-L2370/72+Sb(III) - IOC-L2370/2372-Sb(III) | **5,78402E-09** |
|  | IOC-L2370/2372+Sb(III) - IOC-L2370/2370(C.T.) | 0,147041958 |
|  | IOC-L2370/2372+Sb(III) - IOC-L2372/2372 | 0,641549524 |
|  | IOC-L2370/2372-Sb(III) - IOC-L2370/2370(C.T.) | **2,48142E-08** |
|  | IOC-L2370/2372-Sb(III) - IOC-L2372/2372 | **1,28122E-08** |
|  | IOC-L2370/2370(C.T.) - IOC-L2372/2372 | 0,759602475 |
| **80** | IOC-L2370/2370+Sb(III) - IOC-L2370/2372+Sb(III) | 0,999567155 |
|  | IOC-L2370/2370+Sb(III) - IOC-L2370/2372-Sb(III) | **2,95364E-12** |
|  | IOC-L2370/70+Sb(III) - IOC-L2370/2370(C.T.) | 0,118892055 |
|  | IOC-L2370/2370+Sb(III) - IOC-L2372/2372 | 0,999628126 |
|  | IOC-L2370/72+Sb(III) - IOC-L2370/2372-Sb(III) | **3,1215E-12** |
|  | IOC-L2370/2372+Sb(III) - IOC-L2370/2370(C.T.) | 0,160285272 |
|  | IOC-L2370/2372+Sb(III) - IOC-L2372/2372 | 0,999999999 |
|  | IOC-L2370/2372-Sb(III) - IOC-L2370/2370(C.T.) | **6,14198E-12** |
|  | IOC-L2370/2372-Sb(III) - IOC-L2372/2372 | **3,11506E-12** |
|  | IOC-L2370/2370(C.T.) - IOC-L2372/2372 | 0,158513938 |
| **96** | IOC-L2370/2370+Sb(III) - IOC-L2370/2372+Sb(III) | 0,999832729 |
|  | IOC-L2370/2370+Sb(III) - IOC-L2370/2372-Sb(III) | **2,07464E-10** |
|  | IOC-L2370/70+Sb(III) - IOC-L2370/2370(C.T.) | 0,581730186 |
|  | IOC-L2370/2370+Sb(III) - IOC-L2372/2372 | 0,999991395 |
|  | IOC-L2370/72+Sb(III) - IOC-L2370/2372-Sb(III) | **2,13226E-10** |
|  | IOC-L2370/2372+Sb(III) - IOC-L2370/2370(C.T.) | 0,671070974 |
|  | IOC-L2370/2372+Sb(III) - IOC-L2372/2372 | 0,999223702 |
|  | IOC-L2370/2372-Sb(III) - IOC-L2370/2370(C.T.) | **2,7101E-10** |
|  | IOC-L2370/2372-Sb(III) - IOC-L2372/2372 | **2,04788E-10** |
|  | IOC-L2370/2370(C.T.) - IOC-L2372/2372 | 0,539820855 |
| **104** | IOC-L2370/2370+Sb(III) - IOC-L2370/2372+Sb(III) | 0,999993315 |
|  | IOC-L2370/2370+Sb(III) - IOC-L2370/2372-Sb(III) | **3,12399E-10** |
|  | IOC-L2370/70+Sb(III) - IOC-L2370/2370(C.T.) | 0,831872085 |
|  | IOC-L2370/2370+Sb(III) - IOC-L2372/2372 | 0,999970668 |
|  | IOC-L2370/72+Sb(III) - IOC-L2370/2372-Sb(III) | **3,16884E-10** |
|  | IOC-L2370/2372+Sb(III) - IOC-L2370/2370(C.T.) | 0,863010517 |
|  | IOC-L2370/2372+Sb(III) - IOC-L2372/2372 | 0,999763543 |
|  | IOC-L2370/2372-Sb(III) - IOC-L2370/2370(C.T.) | **3,9218E-10** |
|  | IOC-L2370/2372-Sb(III) - IOC-L2372/2372 | **3,06089E-10** |
|  | IOC-L2370/2370(C.T.) - IOC-L2372/2372 | 0,782175203 |
| **120** | IOC-L2370/2370+Sb(III) - IOC-L2370/2372+Sb(III) | 0,999977117 |
|  | IOC-L2370/2370+Sb(III) - IOC-L2370/2372-Sb(III) | **2,43709E-08** |
|  | IOC-L2370/70+Sb(III) - IOC-L2370/2370(C.T.) | 0,673634908 |
|  | IOC-L2370/2370+Sb(III) - IOC-L2372/2372 | 0,999935752 |
|  | IOC-L2370/72+Sb(III) - IOC-L2370/2372-Sb(III) | **2,31141E-08** |
|  | IOC-L2370/2372+Sb(III) - IOC-L2370/2370(C.T.) | 0,619450913 |
|  | IOC-L2370/2372+Sb(III) - IOC-L2372/2372 | 0,999999824 |
|  | IOC-L2370/2372-Sb(III) - IOC-L2370/2370(C.T.) | **5,06789E-08** |
|  | IOC-L2370/2372-Sb(III) - IOC-L2372/2372 | **2,27544E-08** |
|  | IOC-L2370/2370(C.T.) - IOC-L2372/2372 | 0,603395871 |
| **128** | IOC-L2370/2370+Sb(III) - IOC-L2370/2372+Sb(III) | 0,999991763 |
|  | IOC-L2370/2370+Sb(III) - IOC-L2370/2372-Sb(III) | 1,46806E-05 |
|  | IOC-L2370/70+Sb(III) - IOC-L2370/2370(C.T.) | 0,99876892 |
|  | IOC-L2370/2370+Sb(III) - IOC-L2372/2372 | 0,999943201 |
|  | IOC-L2370/72+Sb(III) - IOC-L2370/2372-Sb(III) | **1,5728E-05** |
|  | IOC-L2370/2372+Sb(III) - IOC-L2370/2370(C.T.) | 0,999667696 |
|  | IOC-L2370/2372+Sb(III) - IOC-L2372/2372 | 0,999618302 |
|  | IOC-L2370/2372-Sb(III) - IOC-L2370/2370(C.T.) | **1,87629E-05** |
|  | IOC-L2370/2372-Sb(III) - IOC-L2372/2372 | **1,31389E-05** |
|  | IOC-L2370/2370(C.T.) - IOC-L2372/2372 | 0,994686018 |
| **144** | IOC-L2370/2370+Sb(III) - IOC-L2370/2372+Sb(III) | 0,98802251 |
|  | IOC-L2370/2370+Sb(III) - IOC-L2370/2372-Sb(III) | **4,75442E-12** |
|  | IOC-L2370/70+Sb(III) - IOC-L2370/2370(C.T.) | 0,96704568 |
|  | IOC-L2370/2370+Sb(III) - IOC-L2372/2372 | 0,716187956 |
|  | IOC-L2370/72+Sb(III) - IOC-L2370/2372-Sb(III) | **4,18665E-12** |
|  | IOC-L2370/2372+Sb(III) - IOC-L2370/2370(C.T.) | 0,999854279 |
|  | IOC-L2370/2372+Sb(III) - IOC-L2372/2372 | 0,927539012 |
|  | IOC-L2370/2372-Sb(III) - IOC-L2370/2370(C.T.) | **4,0179E-12** |
|  | IOC-L2370/2372-Sb(III) - IOC-L2372/2372 | **3,37907E-12** |
|  | IOC-L2370/2370(C.T.) - IOC-L2372/2372 | 0,965074917 |
