# Supplementary material for: Insights from Leishmania (Viannia) guyanensis in vitro behavior and intercellular communication
Source: Parasit Vectors. 2021 Oct 28;14:556. doi: 10.1186/s13071-021-05057-x (PMC8554959; doi:10.1186/s13071-021-05057-x)
Supplement: Supplementary file 7 — Additional file 7: Table S6. Co-culture strategies and density variations under media sharing. [file 13071_2021_5057_MOESM7_ESM.docx]

**Table S6:** Co-culture strategies and densities variations under media sharing.

| **Co-cultured pair** | **Counted strain** | **Maximum density (x10^6^/ml)*** |
| --- | --- | --- |
| - | **2335C** | 20.67 ± 3.06 |
| 2335R | **2335C** | 65.33 ± 2.31 |
| - | **2335R** | 23.50 ± 0.71 |
| 2335C | **2335R** | 15.33 ± 1.53 |
| - | **2370C** | 18.00 ± 1.73 |
| 2372F | **2370C** | 113.33 ± 15.28 |
| - | **2372F** | 10.50 ± 1.50 |
| 2370C | **2372F** | - 1. ± 0.10 |

* Mean and standard deviation are shown.
